# Testing the Specificity of Predictors of Reading, Spelling and Maths: A New Model of the Association Among Learning Skills Based on Competence, Performance and Acquisition

**DOI:** 10.3389/fnhum.2020.573998

**Published:** 2020-12-07

**Authors:** Pierluigi Zoccolotti, Maria De Luca, Chiara Valeria Marinelli, Donatella Spinelli

**Affiliations:** ^1^Department of Psychology, Sapienza University of Rome, Rome, Italy; ^2^Developmental Dyslexia Lab, IRCCS Fondazione Santa Lucia, Rome, Italy; ^3^Lab of Applied Psychology and Intervention, Department of History, Society and Human Studies, University of Salento, Lecce, Italy; ^4^Department of Movement, Human and Health Sciences, Foro Italico University of Rome, Rome, Italy

**Keywords:** comorbidity, reading, spelling, maths, proximal predictors, learning disabilities, dyslexia, acquisition of instances

## Abstract

In a previous study (Zoccolotti et al., 2020) we examined reading, spelling, and maths skills in an unselected group of 129 Italian children attending fifth grade by testing various cognitive predictors; results showed a high degree of predictors’ selectivity for each of these three behaviors. In the present study, we focused on the specificity of the predictors by performing cross-analyses on the same dataset; i.e., we predicted spelling and maths skills based on reading predictors, reading based on maths predictors and so on. Results indicated that some predictors, such as the Orthographic Decision and the Arithmetic Facts tests, predicted reading, spelling and maths skills in similar ways, while others predicted different behaviors but only for a specific parameter, such as fluency but not accuracy (as in the case of RAN), and still others were specific for a single behavior (e.g., Visual-auditory Pseudo-word Matching test predicted only spelling skills). To interpret these results, we propose a novel model of learning skills separately considering factors in terms of competence, performance and acquisition (automatization). Reading, spelling and calculation skills would depend on the development of discrete and different abstract competences (accounting for the partial dissociations among learning disorders reported in the literature). By contrast, overlap among behaviors would be accounted for by defective acquisition in automatized responses to individual “instances”; this latter skill is item specific but domain independent. Finally, performance factors implied in task’s characteristics (such as time pressure) may contribute to the partial association among learning skills. It is proposed that this new model may provide a useful base for interpreting the diffuse presence of comorbidities among learning disorders.

## Introduction

Developmental disorders in reading, spelling and maths tend to be partially associated, a phenomenon known as comorbidity (e.g., Landerl and Moll, 2010). Comorbidity poses an important challenge to classical cognitive models as they are typically focused on accounting for the presence of deficits in a single domain, i.e., reading, spelling or maths (Pennington, 2006). Historically, an impetus to the development of cognitive models (such as the dual route cascade model or DRC in the case of reading; Coltheart et al., 2001) has come from the detailed analysis of selective deficits in reading (spelling or maths) in patients with focal brain lesions. Co-presence of acquired symptoms in patients is common but it can be easily accommodated by positing that the brain lesion has impaired processing in more than a single, distinct brain area (i.e., in terms of anatomical overlap), making associations in the case of acquired problems not particularly interesting. Unlike the case of acquired symptomatology, association among symptoms in the developmental domain is considerably more interesting although difficult to account for.

In a breakthrough analysis of comorbidity, Pennington (2006) has proposed that comorbidity among developmental disorders is to be expected because multiple factors are responsible for developmental disorders, such as dyslexia or ADHD, as they appear at the clinical level, i.e., in terms of “complex behavior disorders”. Importantly, developmental deficits are framed in a multiple level perspective, including the behavioral level (complex behavior disorders), as well the cognitive, the neural and etiological levels. One does not expect 1:1 correspondence between genes, neural structures and cognitive factors, but there are interactions both within and between levels. Thus, a complex behavioral disorder would result from interaction of multiple cognitive factors and comorbidity of behavioral disorders can be explained by such interactions. Albeit quite broad, the multiple level model of Pennington (2006) represents an important reference to frame developmental disorders, in particular since it has the potential to account for both dissociated and associated symptoms.

Indeed, in recent years there has been increasing research aimed to pinpoint the multiple factors underlying the co-presence of developmental disorders. Pennington and colleagues focused on the comorbidity between reading impairment and ADHD. Evidence indicates that children with dyslexia tend to be impaired in phonological tasks while children with ADHD in tasks of inhibition control; however, both groups are also impaired in speed of processing which thus appears as a factor present in both disorders (e.g., Willcutt et al., 2005, 2010). In the same vein, some studies have tried to identify the independent and conjunct factors accounting for the partial overlap between reading and language impairments (Bishop et al., 2009), while others for that between reading and maths (e.g., Slot et al., 2016). Information coming from these studies seems still insufficient to draw definite conclusions. For example, in the case of reading and maths, several different alternatives have been proposed: Slot et al. (2016) proposed that comorbidity is accounted for by phonological processing while Wilson et al. (2015) found common deficits in rapid naming and verbal short-term memory (Wilson et al., 2015). More recently, it was found that children with comorbid dyslexia and dyscalculia presented deficits in visual perception (Cheng et al., 2018). Overall, it seems that up until now studies have failed to converge on a unitary framework (for a discussion on this point, see Astle and Fletcher-Watson, 2020).

It may be observed that research on the cognitive antecedents of comorbidity, such as those above briefly reported, have been largely developed outside the traditional models of cognitive models of reading spelling and maths. Indeed, as stated above, cognitive architectures postulated by cognitivist models (extended to developmental disorders, but originally derived from studies in patients with acquired symptoms) seem to offer limited information to explain comorbidity of learning disorders as they are typically focused in explaining single behaviors (e.g., reading or maths).

So, one can draw a fairly clear distinction between studies that tried to isolate the cognitive antecedents of the co-morbidity of two (or more) developmental disorders which typically only loosely referred to the existing cognitive models for these disorders and studies, framed within the cognitivist tradition, that typically aimed to account for deficits within a single deficit perspective (e.g., reading or maths), generally selecting participants to the studies according to a single-deficit category (Peters and Ansari, 2019). It should be noted that there is some overlap in the cognitive processes these two lines of research refer to. So, for example, phonological processes are invoked both within the co-morbidity approach in accounting for the presence of dyslexia (along with perceptual speed, Willcutt et al., 2005, 2010) and within cognitive models of reading (such as the triangle model, Plaut et al., 1996) to account for at least some proportion of the deficits in reading. Still, the perspective in which cognitive processes are referred to is quite different in the tradition of cognitive architectures and in the recent comorbidity studies focusing on the overlap between cognitive processes. In the first case, there is an explicit attempt to express the nature of the relationship between the cognitive process and the target behavior within a given architecture (e.g., the cognitive model of reading, spelling, or maths). Thus, a cognitive architecture is a complex model in which all the interactions between the involved processes, necessary to account for a given target behavior, are made explicit. In this perspective, cognitive predictors may be described as the “proximal” factors accounting for the performance (as well as different forms of impairment) in a given behavior (Coltheart, 2015). By contrast, cognitive processes have been referred to as “distal” if some relationship between the process and the target behavior is expected (and empirically proven), but its nature is not made explicit as well as its relationships with the other cognitive processes contributing to predict a given behavior. Coltheart (2015) describes distal factors by stating that they affect behavior indirectly by influencing the proximal factors in the model. In this vein, one can easily see that short-term memory, attention and the like are important for efficient reading but their action would influence the target behavior by modulating the activity of the proximal factors envisaged by the model (e.g., memory may modulate the application of phoneme-grapheme rules in the example provided by Coltheart, 2015). Note that cognitive skills are neither proximal nor distal as such; rather, this distinction refers to the way in which one looks at a given cognitive factor (for a thorough discussion see Coltheart, 2015). Overall, research on the cognitive antecedents of comorbidity has moved away from referring to cognitive models as they are typically focused in explaining single behaviors (i.e., reading, spelling or maths) and, with few exceptions (see below), offer no explicit base to develop predictions for explaining the widespread presence of comorbidity among developmental disorders.

There is another, critical reason why research on comorbidity is difficult to frame within cognitive architectures. Cognitive models typically aim to account for behaviors (e.g., reading or maths) seen in highly abstract forms. For example, the DRC (Coltheart et al., 2001) spells out the processes for reading aloud single mono-syllabic words in English. It is only by extrapolation that this model may be used to account for the performance in actual, common reading tasks, such as silently reading a text. In other terms, models such as the DRC aim to account for the competence that is hypothesized to underlie the ability (as well as the derangement) in a given domain, not the actual behavior in naturalistic conditions (for a discussion see Bishop, 1997). So, cognitive models generally fail to describe (or leave largely underspecified) the processing factors through which actual performance can be explained (Bishop, 1997). In Chomsky (1966) terms, cognitive architectures describe “competence” factors but are silent as to the “performance” factors involved. By contrast, in the example of reading, it is likely that naturalistic conditions (such as the presence of multiple words and lines in the text; the necessity of moving the eyes from a word to the next; the memory load involved when fixating and processing the next word while pronouncing the preceding word) involve performance factors which are not the same of those in reading single short words. Attention to performance factors is consistent with Pennington (2006) view that proposes that comorbidity occurs among “complex behavioral disorders”. Accordingly, an analysis focusing only on “competence” factors may indeed fail to provide a full account for the actual behavioral disorders.

However, we propose that the approach based on cognitive architectures may be adjusted to account for the presence of comorbidities among developmental disorders. Indeed, in a few cases, this has been done. For example, it has been proposed that reading and spelling may rely on the same lexicon (Allport and Funnell, 1981; Coltheart and Funnell, 1987; Behrmann and Bub, 1992; Angelelli et al., 2010a). Even though this proposal is controversial and alternative options based on the idea of multiple lexica have been proposed (for a discussion, see Hillis and Rapp, 2004), this case provides an interesting demonstration that, in principle, cognitive architectures may be developed which explicitly consider more than a single behavior.

## Aim of the Study

In the present report, as well as a previous companion one (Zoccolotti et al., 2020), we aimed to develop a unitary model to uncover the unique and shared influences of predictors for reading, spelling and maths skills. To this aim, we examined these performances in an unselected group of fifth grade Italian children. It is well known that performances in reading (spelling or maths) can be described on a continuum such that so-called pathological performances merely reflect low points on a continuous distribution (e.g., Protopapas and Parrila, 2018). So, we considered as an appropriate starting point to examine performances within an unselected sample of children, though the ultimate goal of this work is to develop a model able to account for the comorbidities of learning disorders, and in particular for both the presence of associations and dissociations among them.

The present report is strictly linked to a previous one based on the same dataset (Zoccolotti et al., 2020), which presented the following main features. First, as target dependent behaviors we selected ecological tasks, such as reading a text passage, spelling a passage under dictation, and making calculations. Second, we used a “proximal” approach, that is we formulated explicit causal relationships between predictors (i.e., cognitive antecedent of the specific behavior under scrutiny) and target dependent measures (i.e., reading, spelling or maths). Third, based on the relevant literature, predictors of reading, spelling and maths were selected pointing to both efficacy and parsimony (details of such a selection are given in Zoccolotti et al., 2020). Fourth, as a control, we tested the possible predictive power of general cognitive dimensions (i.e., measures of short-term memory, phonemic verbal fluency, visual perceptual speed, and non-verbal intelligence). These can be seen as “distal” predictors in the sense that some relationships with the dependent measures are expected but the nature of these relationships is not specified, and they may occur through complex interrelationships with the cognitive proximal predictors.

**FIGURE 1:**
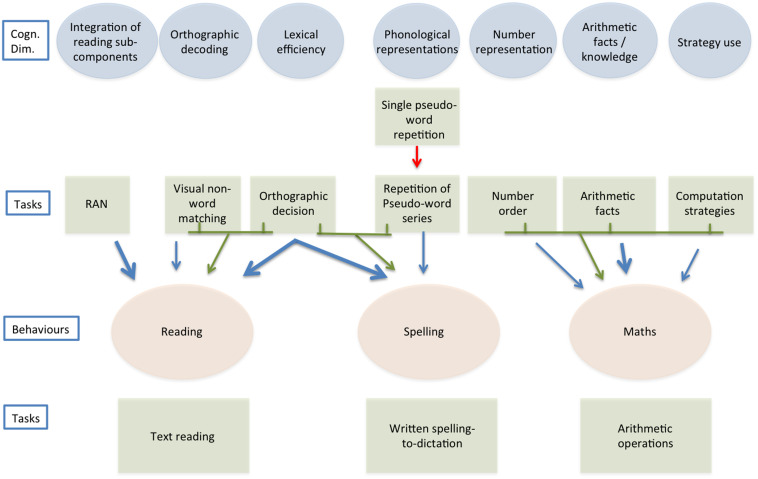
Predictors of reading, spelling, and doing maths (based on the results from Zoccolotti et al., 2020). The figure presents the main links observed between tasks, used as predictors, and reading (fluency), spelling, and maths (accuracy) measures. Both direct links (in blue) and links expressing communalities (green arrows coming from green lines connecting square boxes) between predictors are reported (for the sake of presentation only communalities with beta of ca.05 or more are reported). The “heavier” blue arrows indicate “strong” influences (i.e., beta of ca.10 or more). The red arrow under the Single pseudo-word repetition box indicates a suppressive effect.

Based on communality analyses, presented analytically in Zoccolotti et al. (2020), we developed separate models to account for the abilities to read, spell and do maths. All these models explained a sizeable amount of variance (ranging from 27.5% in the case of calculation accuracy to 48.7% of reading fluency). The only exception was reading accuracy for which the models based on specific and general factors yielded similarly limited results (for this reason, reading accuracy will not be considered in the present report). Figure 1 synthesizes the conclusions of the previous study, and is the starting point of the present one. Figure 1 presents schematically these models illustrating the cognitive dimensions used as predictors and the target behaviors (as well as the tasks used to measure both). Models based on general cognitive factors also accounted for some variance (ranging from 6.5% in the case of writing to 19.5% in the case of reading fluency) but this was appreciably less than that explained by models based on the hypothesized proximal predictors. Furthermore, when general predictors were added one by one to models based on specific predictors, in most cases they did not add unique variance while they accounted for some shared variance with other variables, and the overall increase in explained variance was in most cases very small (for these analyses please refer to Supplementary Table 2 in the Supplementary Materials, Zoccolotti et al., 2020^1^).

Overall, the models of reading, spelling and maths proposed in Zoccolotti et al. (2020) and summarized here in Figure 1 can be considered “specific” because, using sets of predictors which mark different putative dimensions for different behaviors, they explained a relevant amount of variance in each of these behaviors. Furthermore, they showed greater efficacy than models based on general cognitive factors (i.e., distal predictors in Coltheart’s terms). Still, only a limited amount of variance was explained by shared factors. The Orthographic Decision test (see Zoccolotti et al., 2020) worked as a predictor of both reading and spelling, a finding consistent with the literature, which indicates that a single orthographic lexicon may account for performance in reading and spelling (Allport and Funnell, 1981; Coltheart and Funnell, 1987; Behrmann and Bub, 1992; Angelelli et al., 2010a). However, apart from this, models for different skills were based on different factors. In particular, the factors selected to predict maths were entirely separate from those of reading and spelling. This selectivity is not surprising as, up until now, it has proven difficult to pinpoint the factors which account for the co-morbidity between reading and calculation disorders (e.g., Wilson et al., 2015; Slot et al., 2016; Cheng et al., 2018). One proposal that has been advanced is that phonological skills may account for such co-morbidity (Slot et al., 2016). Our original analyses did not offer much support to this proposal; in fact, phonological skills provided a relevant contribution only to predict spelling (Zoccolotti et al., 2020).

In the present report, we submit to a more stringent test the conclusion that the models of reading, spelling and maths reported by Zoccolotti et al. (2020) are indeed “specific”. This was done by using predictors in a cross-over manner, that is evaluating whether predictors of a “target” behavior (e.g., skill in arithmetical facts predicting maths) also had an influence on “non-target” behaviors (e.g., skill in arithmetical facts predicting spelling or reading). Our working hypothesis was that, if models are specific, predictors used in such a cross-over manner should fail to make effective predictions. Thus, for example, one should expect that the set of predictors in the model of reading fluency would not predict spelling or maths or, possibly, that they would predict such behaviors in ways that cannot be distinguished by the model based on general cognitive abilities. Conversely, one should expect that predictors in the model of maths would not predict reading and spelling and so on. The results of the present study were used to develop a model aimed to account for both the overlap and the dissociation between learning skills (and deficits).

## Materials and Methods

### Participants

An unselected sample of 129 (65 male, 64 female) Italian children (mean age = 10.7 years; SD = 0.3; range = 10.1-11.3 years) participated in the study. These participants are the same as in Zoccolotti et al., 2020. All children attended fifth grade in two schools in Rome and three in Latina in low-middle social class environments. Children from a total of 14 classes participated in the study. All children had an adequate performance to the Raven CPM (Pruneti et al., 1996).

Parents were informed about the screening activities and authorized their child’s participation by signing the appropriate informed consent paperwork. The study was carried out in accordance with the principles of the Helsinki Declaration and was approved by the ethical committee of the IRCSS Fondazione Santa Lucia and by the school authorities.

### Materials

Following is a brief description of the tests used, divided between dependent measures, putatively specific predictors and general cognitive predictors (for a more in-depth description of the test materials, please refer to Zoccolotti et al., 2020).

#### Dependent Measures for Reading, Spelling, and Maths

##### MT reading test (Cornoldi and Colpo, 1998)

The child reads a text passage aloud with a 4-min time limit; reading time (in seconds per syllable) and accuracy (number of errors) are scored.

##### “Nonna Concetta” spelling-to-dictation (Marinelli et al., 2016a)

The child has to spell a text dictated by an examiner, including both consistent and inconsistent words. Therefore, the task cannot be solved exclusively through sub-lexical phoneme-to-grapheme mapping, but requires the retrieval of lexical representation also in a consistent orthography such as Italian. The total number of elements for which a mistake is present is scored.

##### Mental and written arithmetic calculations subtests (from the test AC-MT 6-11; Cornoldi et al., 2002)

As to mental calculations, the child performs three sums and three subtractions of two two-digit numbers in the mind as quickly and as accurately as possible. The percentage of errors is scored. The time to perform the task is scored only to stop the task if 30” per calculation are elapsed. As to written calculations, the child performs two calculations for each of the four basic number operations, based on two numbers. The percentage of errors is calculated. An “*accuracy score”* derived from both the mental and written arithmetic calculations and a “*time score,”* derived only by the written calculation test, were used as dependent measures.

#### Specific Cognitive Predictors

##### RAN (De Luca et al., 2005)

The child is shown matrices of colored squares or digits and is requested to name each stimulus as quickly and accurately as possible. The time to complete the task is measured (in seconds per item).

##### Orthographic decoding (Visual-auditory pseudo-word matching test)

In this test (specifically devised for this project on comorbidity), the child has to say whether or not two pseudo-words presented in the visual modality, in a mixed visual-auditory modality or in the auditory-auditory presentation are the same or not. Accuracy is measured as percentage of errors. The *Visual-visual and Auditory-auditory* presentations were used in the original report but are not presented here as they did not enter in the original models.

##### Orthographic decision

In this test (specifically devised for this project), the child has to silently read a list of high- and low-frequency inconsistently spelled words (and corresponding derived pseudo-homophones, homophonic to the original words but orthographically incorrect for the presence of a phonological plausible error) and to indicate whether or not they are correctly spelled. Then the task taps the retrieval of the orthographic representation thought lexical reading. The percentage of errors in judging both correct words and pseudo-homophones is scored.

##### Single pseudo-word repetition and phonemic segmentation

In this test (specifically devised for this project), thirty long pseudo-words were presented in the auditory modality. The child has to repeat each stimulus and, then, segment it by phonemes. The percentages of correct repetitions and correct segmentations were scored. Only data relative to the Single Pseudo-word Repetition part of the test are used in the present report.

##### Repetition of pseudo-word series (Marinelli et al., 2020b)

The child is asked to listen to 10 series of triplets of pseudo-words and repeat the items of each series in the same order immediately after the acoustic warning. The percentage of correct repetitions was used in the analysis.

##### Number order test (from the AC-MT 6-11 battery; Cornoldi et al., 2002)

The child has to order 10 series of 4 numbers. The percentage of wrong series was entered into the analyses.

##### Arithmetic facts test (from the developmental dyscalculia battery; Biancardi and Nicoletti, 2004)

The child has to say the result of a series of multiplications as rapidly as possible. The percentage of incorrect responses (taking into consideration incorrect response as well as response given beyond time limit or attempts based on the use of a mental calculation) is scored.

##### Computation strategies test (from the AC-MT 11-14 battery; Cornoldi and Cazzola, 2003)

The child must determine the result of arithmetic operations without actually calculating them, but reasoning on the base of similar complete calculations that are shown beside. The percentage of computations performed correctly within the time limit was used in the analyses.

The following tests (maths domain) were also administered but they did not enter in the original models and they are not referred to in the present report: Computation Procedures (Tabulation and carry); Backward Counting (from the AC-MT battery; Cornoldi et al., 2002); Dictation of Numbers (from the AC-MT battery; Cornoldi et al., 2002); Arabic Number Reading test (from the Developmental Dyscalculia Battery; Biancardi and Nicoletti, 2004).

#### General Cognitive Predictors

Performance in the following general cognitive tests was considered for the control models. The putative target dimension is presented in brackets:

##### Symbol search subtest (subtest from the WISC-R; Wechsler, 1986) (cognitive speed)

The child has to mark a box if a string of symbols contains one or both of the symbols presented on the left of the string, working as rapidly as possible. The percentage of correct responses out of the trials performed within 2 minutes was used.

##### Raven’s colored progressive matrices (non-verbal intelligence)

The percentage of correct responses was scored and used for the analyses.

##### Forward and backward span of numbers (from the BVN battery; Bisiacchi et al., 2005) (verbal short-term memory)

The forward task requires the immediate serial recall of a sequence of digits. The span corresponds to the last length for which at least two sequences were correctly recalled. In the backward task the child has to recall each sequence in backward order. The forward and backward spans were measured.

##### Verbal phonemic fluency test (from the BVN battery, Bisiacchi et al., 2005) (verbal fluency)

The child has to generate as many words as possible from the initial letters C, S, and P in a minute. The number of correct items is scored.

#### Procedure

Children were tested in a quiet room in their schools. Three examiners examined approximately a third of the sample each. To insure homogeneity of administration, examiners participated to an intensive training before the study with the supervision of one of the authors, MDL).

Most tests were performed individually, while a few (*Written Arithmetic Calculations, Number Order and Raven’s Colored Progressive Matrices*) were collectively administered to small groups of children. About three hours of testing were necessary to complete the battery. Most children completed testing in 3 sessions.

Order of tests was fixed and was the following (in brackets are tests which were administered but are not considered in the present report): MT reading test, RAN test, “Nonna Concetta” Spelling-to-dictation test, Verbal Phonemic Fluency test, Mental Calculation, (Backward Counting), (Dictation of Numbers), Forward and Backward Span of Numbers, Raven’s Colored Progressive Matrices, Number Order test, Written Arithmetic Calculations, Symbol Search subtest, Repetition of Pseudo-word Series, (Arabic Number Reading test), Orthographic Decision test, Arithmetic Facts test, Single Pseudo-word Repetition and Phonemic Segmentation tests, Computation Strategies test, (Computation Procedures, Tabulation and carry test), Orthographic decoding: (Visual-visual), Visual-auditory Pseudo-word Matching test (Auditory-auditory).

### Data Analysis

Descriptive statistics (N, mean, SD, coefficient of variation, min and max values observed, maximum values maximum possible score - only in the case of closed scales -, and reliability values) are presented in Supplementary Table 1 in the Supplementary Materials. Furthermore, Supplementary Table 2 in the Supplementary Materials reports the Pearson intercorrelations among all variables.

The results are based on the commonality analysis, a method of variance partitioning designed to identify proportions of variance in the dependent variable that can be attributed uniquely to each of the independent variables, and proportions of variance that are attributed to various combinations of independent variables (Pedhazur, 1982; Nimon, 2010). Notably, some of these interactions might also reveal suppressive effects, i.e., in the cases in which the predictor shares variance with another predictor and this variance does not contribute directly to performance on the dependent measure.

Communality analysis is a powerful analysis that is most effective in the case of a limited set of predictors. This feature is useful here since our general aim is to build models of performance characterized by both effectiveness (in terms of total variance explained) and parsimony (in terms of number of predictors used).

In our first report (Zoccolotti et al., 2020), communality analyses were used for identifying the most effective models of reading, spelling and maths. Here, our focus was in testing the specificity of the models originally developed for reading, spelling and maths. If the original models were indeed specific, testing predictors over non-target behaviors should fail to effectively predict target behaviors. These hypotheses were tested by switching predictors over dependent measures; thus, we checked to what extent the predictors of reading accounted for spelling and calculation and so on.

To anticipate, these analyses indicated that set of predictors exerted a significant influence also over non-target behaviors. To further understand these patterns we examined the relative efficacy of each specific predictor over both target and “non-target” behaviors, also by separately adding them into each of the original models. An analytic description of this procedure is provided in the section “RESULTS.”

## Results

First, we present an overview of the communality analyses run on non-target behaviors and how they compare in terms of general explanation (R^2^) to the original models of reading, spelling and maths as well as to the model based on general cognitive predictors (for more information on how these models were devised and tested please refer to Zoccolotti et al., 2020). The outcome of these analyses is summarized in Table 1 which presents the total variance accounted for by using the predictors in the models of reading, spelling, and maths when applied to the target as well as all non-target behaviors (the list of predictors used in the original analyses is also reported in Part A of the table). Inspection of the table shows that each set of “specific” predictors always yields the highest estimate on the target behavior (i.e., direct models). Thus, reading fluency is best accounted for by the predictors in the reading model (Orthographic Decision, RAN, and Visual-auditory Pseudo-word Matching) and the same holds true for spelling (predictors: Orthographic Decision, Single Pseudo-word Repetition and Repetition of Pseudo-word Series) and calculation, both speed and accuracy (predictors: Number Order, Arithmetic Facts, and Computation Strategies).

**TABLE 1:** Part A) Predictors in the original models of reading, spelling, and maths and in the general cognitive factors model (from Zoccolotti et al., 2020). Part B) Percentage of total variance explained by different models.

PART A					

	Original models	Reading fluency model	Spelling accuracy model	Calculation (fluency and accuracy) model	General cognitive factors model
	Predictors	- Orthographic Decision - RAN - Visual-auditory Pseudo-word Matching	- Orthographic Decision - Single Pseudo-word Repetition - Repetition of Pseudo-word Series	- Number Order - Arithmetic Facts - Computation Strategies	- Raven - Symbol Search - Backward Span - Verbal Phonemic Fluency

**PART B**					

**Dependent measures**	Reading fluency	**48.7**	34.9	38.8	19.5
	Spelling accuracy	21.1	**29.2**	18.6	6.5
	Calculation speed	31.9	18.1	**37.9**	12.8
	Calculation accuracy	19.4	20.2	**27.5**	12.8

*In particular, each specific set of predictors is used to predict the target behavior as well as all the other non-target behaviors. The variances explained by the “specific” predictions (i.e., reading predicted by predictors in the reading fluency model etc.) are marked in bold. For comparison, the variances accounted for by the general cognitive factors model are also presented.*

However, inspection of the table also clearly illustrates that putatively specific models predict much more than one would expect (and much more than what accounted for by general cognitive predictors) of the other “non-target” dependent behaviors. Thus, the predictors of the reading model account for 21.1% of the total variance in spelling, 31.9% of the variance in calculation (speed) and 19.4% of that in calculation (accuracy). Much the same occurs when using predictors of spelling and calculation. In fact, some of the values are particularly high. For example, the predictors in the model of calculation account for 38.8% of the variance in reading fluency, a value only slightly inferior to the variance predicted by the model of reading itself (48.7%) and even higher than the two specific models of calculation (which accounted for 37.9% and 27.5% of variance for time and accuracy, respectively; see Table 1).

Notably, all values for predictions over non-target behaviors are appreciably higher than those of the model based on general cognitive factors (i.e., predictors: Raven, Symbol Search, Backward Span, Verbal Phonemic Fluency; see last column of Table 1). A further test on the possible role of the general predictors was carried out by replicating the communality analyses using as dependent variables the standardized residuals once the effect of the general cognitive factors was partialled out (based on multiple regression analyses). These results are summarized in Supplementary Table 3 in the Supplementary Materials. Even with this stringent test, the described pattern holds although partially attenuated; thus, for example, the predictors of the reading model account for 38.9% of the model of reading but also 18.0% of the variance in spelling, 25.2% of the variance in calculation (speed) and 12.9% of that in calculation (accuracy).

The absolute level of variance differs somewhat among behaviors. Some of these differences may be due to different levels of reliability of the dependent measures. Thus, reliability values tend to be generally higher for reading fluency than for the other measures. To normalize data with respect to this critical aspect, in Table 2, values of total explained variance are expressed in terms of “true” score variances, i.e., the product of observed score variance and reliability of the test (but see Kang and MacDonald, 2010, for limitations on this procedure).

**TABLE 2:** Percentage of “true variance” explained by models based on different sets of predictors i.e., total variance values adjusted for the reliability values of the four dependent measures.

		Predictors			
		Reading fluency model	Spelling accuracy model	Calculation (fluency and accuracy) model	Cognitive abilities model
**Dependent measures**	Reading fluency	**57.3**	41.1	45.6	22.9
	Spelling accuracy	28.1	**38.9**	24.8	8.7
	Calculation speed	40.9	23.2	**48.6**	16.4
	Calculation accuracy	38.8	40.4	**55.0**	25.6

*The reliability values were the following: Reading fluency: *r* = 0.85; Spelling accuracy: *r* = 0.75; Calculation speed = 0.78; calculation accuracy = 0.50. The true variance explained by the “specific” predictions (i.e., reading dependent measure predicted by reading predictors etc.) are marked in bold.*

After correction for reliability differences among dependent measures, values of total true variances are somewhat less different from each other. Still, it is clear that putatively “specific” predictors tend to have strong influences across different learning processes, well beyond the values observed in the case of general cognitive predictors.

The communality analyses are presented more extensively in Table 3 in terms of both coefficients and percentage of explained variance for each factor separately and in common with the others in the model. We also examined the 95% confidence limits of the coefficients; these estimates were obtained as accelerated bootstrap confidence intervals produced over 1,000 iterations (Nimon and Oswald, 2013). These analyses are graphically presented in Supplementary Figures 1–4 of the Supplementary Materials.

**TABLE 3:** Cross-analyses carried out by switching predictors over dependent measures: predictors in the models of reading, spelling, and calculation are used to test whether they also predict non-target behaviors.

	A.Reading (fluency)		B.Spelling		C.Calculation (speed)		D.Calculation (accuracy)	
	Coeff.	% *R*^2^	Coeff.	% *R*^2^	Coeff.	% *R*^2^	Coeff.	% *R*^2^
**Predictors in the Reading fluency model**								
Unique to RAN	**0.12**	**24.5**	0	0.3	0.13	40.6	0	0.0
Unique to Orthographic Decision (OD)	**0.19**	**39.3**	0.2	95.4	0.13	39.3	0.12	59.8
Unique to Visual-auditory Pseudo-word Matching (V-ApwM)	**0.03**	**6.9**	0.01	2.5	0	1.3	0.02	9.9
Common to OD and RAN	**−0.02**	**−4.2**	0	1.3	**−**0.02	**−**5.4	0	0.3
Common to V-ApwM and RAN	**0.04**	**8.3**	0	0.6	0.02	6.1	0	0.6
Common to OD and V-ApwM	**0.10**	**19.9**	0	1.5	0.04	11.5	0.06	29.5
Common to OD and RAN and V-ApwM	**0.03**	**5.3**	0	**−**1.5	0.02	6.6	0	**−**0.1
Total	**0.487**		0.211		0.319		0.194	
**Predictors in the Spelling model**								
Unique to Orthographic Decision (OD)	0.16	45.0	**0.12**	**41.3**	0.13	71.1	0.1	48.0
Unique to Single Pseudo-word Repetition (SpwR)	0.01	1.5	**0.06**	**19.5**	0.01	7.9	0	1.0
Unique to Repetition of Pseudo-word Series (RpwS)	0.03	8.5	**0.07**	**23.6**	0	0.7	0.03	14.0
Common to OD and SpwR	0	0.7	**−0.01**	**−2.2**	0	2.1	0	**−**0.5
Common to OD and RpwS	0.08	23.6	**0.1**	**34.7**	0.01	4.7	0.06	30.0
Common to RpwS and SpwR	0.02	6.1	**−0.04**	**−12.9**	0	**−**0.5	0	**−**0.5
Common to OD and SpwR and RpwS	0.05	14.6	**−0.01**	**−4.1**	0.03	14.1	0.02	7.9
Total	0.349		**0.292**		0.181		0.202	
**Predictors in the Calculation model**								
Unique to Number Order (NO)	0	0.5	0.05	27.8	**0.00**	**0.0**	**0.03**	**9.7**
Unique to Arithmetic facts (AF)	0.16	40.3	0.03	15.0	**0.19**	**49.4**	**0.04**	**15.4**
Unique to Computation strategies (CS)	0.09	23.9	0.01	6.0	**0.05**	**12.6**	**0.06**	**22.1**
Common to NO and AF	0	0.6	0.03	14.5	**0.02**	**4.3**	**0.02**	**8.6**
Common to NO and CS	0	0.2	0.02	11.7	**0.01**	**1.7**	**0.04**	**12.7**
Common to CS and AF	0.08	20.5	0.01	6.3	**0.07**	**17.1**	**0.03**	**12.1**
Common to NO and AF and CS	0.05	14.0	0.03	18.7	**0.056**	**14.8**	**0.054**	**19.5**
Total	0.388		0.186		**0.379**		**0.275**	

*For each predictor, the overall standardized β coefficient and percentage of variance explained in the communality analysis (% *R*^2^ = Total/*R*^2^) are reported. Results for model predictions over target behaviors (i.e., set of predictors of reading predicting reading fluency etc.) are reported in bold.*

Table 3 allows examining the efficacy of each predictor (singly and/or in common with others) in contributing to the cross-over tests. It may be noted that some of the predictors show a very high efficacy in the cross-over tests while others show a more selective influence. Below we describe the influence of each predictor analyzing the breadth of its impact across different behaviors. We also note whenever different predictors appear to have overlapping influences across target and non-target behaviors.

### Orthographic Decision and Arithmetic Facts Tests

Inspection of Table 3 shows that performance in the Orthographic Decision test does not only predict performance in reading and in spelling, but also strongly enters in the prediction of calculation skills. Indeed, it accounts for 39.3% of the unique variance in the case of calculation speed with a β of.12 and 59.8% of the unique variance in the case of calculation accuracy with a β of.12. A very similar pattern is observed in the case of the Arithmetic Facts test. This latter is a strong predictor of calculation skills (particularly in the case of calculation speed) but it is also a strong predictor of reading fluency (β = 0.16, 40.3% of accounted variance) and also, although to a lesser extent, of spelling (β = 0.03, 15.0% of explained variance). It seems that the variance accounted for by these two tests (Orthographic Decision and Arithmetic Facts) is similar. To directly check this impression, we run analyses in which we added only the Arithmetic facts test to the original “Reading” and “Spelling” models. These results are illustrated in Table 4; the table also presents similar analyses carried out with all the other predictors considered (results are illustrated below).

**TABLE 4:** Changes to various original models (from Zoccolotti et al., 2020) when a new predictor is added.

Added predictor	Original Model	*% R^2^* original model	*% R^2^* with the added predictor	Un.	Com.	Tot.	% *R*^2^ Tot	% *R*^2^ Un.	Variance shared with
Arithmetic Facts (AF)	Reading (fluency)	48.7	48.9	0.003	0.29	0.29	59.7	0.6	OD (14%); OD and V-ApwM (16%)
	Spelling	29.2	30.9	0.02	0.08	0.10	32.7	5.7	OD (18%); OD and SpwR (16%)
Orthographic Decision (OD)	Calculation (speed)	37.9	38.2	0.004	0.16	0.17	43.4	1.0	AF (12%)
	Calculation (accuracy)	27.5	28.9	0.01	0.16	0.17	60.2	4.7	NO and AF and CS (16%)
RAN	Calculation (speed)	37.9	40.2	0.02	0.13	0.15	38.0	5.8	AF (23%)
	Calculation (accuracy)	27.5	29.1	0.02	−0.01	0.002	0.6	5.4	–
	Spelling	29.2	29.2	0.000	0.001	0.001	0.5	0.03	–
Computation Strategies (CS)	Reading (fluency)	48.7	53.4	0.05	0.18	0.23	42.6	8.9	OD (18%)
	Spelling	29.2	29.6	0.004	0.08	0.08	26.9	1.2	OD (10%); OD and SpwR (15%)
Repetition of Pseudo-word Series (RpwS)	Reading (fluency)	48.7	50.1	0.01	0.17	0.18	36.8	2.8	OD (10%)
	Calculation (speed)	37.9	38.5	0.01	0.03	0.03	8.9	1.6	–
	Calculation (accuracy)	27.5	28.6	0.01	0.09	0.10	36.6	3.6	NO and AF and CS (11%)
Visual-auditory Pseudo-word Matching (V-ApwM)	Calculation (speed)	37.9	37.9	0.000	0.08	0.08	21.5	0.1	AF (11%)
	Calculation (accuracy)	27.5	28.6	0.01	0.07	0.08	27.0	4.0	–
	Spelling	29.2	29.6	0.004	0.007	0.01	2.2	1.2	–
Number Order (NO)	Reading (fluency)	48.7	48.8	0.001	0.06	0.06	12.1	0.3	–
	Spelling	29.2	30.8	0.02	0.12	0.14	43.8	5.3	OD (16%); OD and SpwR (21%)
Single Pseudo-word Repetition (SpwR)	Reading (fluency)	48.7	48.8	0.001	0.08	0.08	16.4	0.03	–
	Calculation (speed)	37.9	37.9	0.000	0.04	0.04	11.2	0.000	–
	Calculation (accuracy)	27.5	27.6	0.001	0.02	0.02	5.9	0.02	–

*The first column indicates the added predictor; the second the original model; next, the total percentage of variance (*R*^2^) explained by the original model is presented (from Zoccolotti et al., 2020), followed by the indication of the total variance (*R*^2^) explained by the same model after the addition of the predictor. Then, unique, common and total contributions of each added predictor are presented (as raw beta coefficients). The next columns report the% of total and unique variances (*R*^2^) accounted by the added predictor with respect to the total variance explained by the model. The last column reports the list of predictor(s) with which the added predictor shared variance (in excess of 10%).*

The total variance accounted in reading fluency changes minimally when the Arithmetic Facts test is added to the predictors of the original Reading model (passing from 48.7% to 48.9%, Table 4). This is actually a general finding as this occurs for all variables included in Table 4 but one (which will be commented later); thus, this result will not be repeated in the text for each variable. The Arithmetic Facts test contributes minimally in terms of unique variance (β = 0.003, 0.6% of accounted variance) but substantially in terms of shared variance (β = 0.29), most of which was with the Orthographic Decision test (see last column of Table 4 which reports the predictor(s) with which the added predictor shared at least 10% of variance). As stated above, the total percentage of *R*^2^ provides an estimate of its overall influence summing its unique contribution and that shared with other variables. Overall, the Arithmetic Facts test contributes for a quite substantial amount of the explained variance of the model of reading fluency (*R*^2^ = 59.7%).

Results are similar in the analysis on spelling. When the Arithmetic Facts test is added to the predictors in original spelling model, it contributes little in terms of unique variance (β = 0.02, 5.7% of accounted variance) but more substantially in terms of shared variance (β = 0.08), most of which was with the Orthographic Decision test (see last column of Table 4). Overall, the Arithmetic Facts test contributes appreciably to the explained variance of the model (*R*^2^ = 32.7%).

Then, we carried out a similar analysis with the Orthographic Decision test on the calculation skills; namely, we added this test to the predictors in the original models of “calculation speed” and “calculation accuracy”. In the former case (Table 4), the Orthographic Decision test does not account for unique variance (β = 0.004, 1.0% of accounted variance) but it appreciably contributes to shared variance (β = 0.16) much of which was with the Arithmetic Facts test (see last column of Table 4). Overall, the Orthographic Decision test contributes appreciably to the explained variance of the model of calculation speed (*R*^2^ = 43.4%). Similar results were obtained when adding the Orthographic Decision test to the predictors for calculation accuracy (Table 4). The Orthographic Decision test contributes minimally in terms of unique variance (β = 0.01, 4.7% of accounted variance) but much more so in terms of common variance (β = 0.16), shared with the Arithmetic Facts, the Number Order and Computation Strategies tests. Overall, the Arithmetic Facts test contributes substantially to the explained variance of the model of calculation accuracy (*R*^2^ = 60.2%).

Overall, these data indicate that, in spite of their surface differences, the Orthographic Decision and the Arithmetic Facts tests exert similar influences across reading, spelling and maths.

### RAN Test

Other predictors show a pattern of both associations and dissociations. In the original models (Zoccolotti et al., 2020), the RAN test was a strong predictor of reading fluency (but not accuracy) contributing with both unique and shared variance to the overall prediction; by contrast, RAN did not contribute to the model of spelling. Thus, there was an indication that RAN contributed to measures of time but not accuracy. Much the same occurs when the RAN task is used in the present study as a predictor of calculation skills (see Table 3). It is a strong predictor of unique variance in the case in which time is measured (β = 0.13, 40.6% of explained variance) while it does not contribute at all (β = 0.00) in the case in which calculation accuracy is considered (Table 3).

When the RAN test is added to the model of calculation speed (see Table 4), it does not account for much unique variance (β = 0.02, 5.8% of accounted variance) but it appreciably contributes to shared variance (β = 0.13) much of which was with the Arithmetic Facts test (see last column of Table 4). Overall, the RAN test contributes substantially to the explained variance of the model of calculation speed (*R*^2^ = 38.0%). By contrast, the contribution of RAN to the model of calculation accuracy is minimal both in terms of unique variance (β = 0.02, 5.4%) as well as shared variance (β = −0.01) and the overall contribution to the variance of the model was negligible (*R*^2^ = 0.6%). Also in the case of spelling, no increase of explained variance was present when the RAN test was added to predictors, and the contribution of this variable was negligible both in terms of unique and common variance.

Overall, these data indicate that the RAN task contributes to the prediction in the case of dependent measures based on time but not in the case of dependent measures based on accuracy.

### Computation Strategies Test

The Computation Strategies test also shows an interesting pattern of co-associations (see Table 3). In particular, it entered in both models of calculation speed and accuracy. However, it also strongly predicted unique variance in reading fluency (β = 0.09, 23.9% of explained variance) and, to a lesser extent, in spelling accuracy (β = 0.01, 6.0% of explained variance).

To understand the possible relationship between performance in this test and the other reading markers we added the Computation Strategies test to the predictors of the original Reading model (Zoccolotti et al., 2020). The results of this analysis (see Table 4) are quite surprising. Indeed, adding the Computation Strategies test appreciably increases the total power of the model passing from the original 48.7% to 53.4%, indicating a total increase in explanatory power of 4.7%. The Computation strategies test contributes both unique (β = 0.05; 8.9% of the total variance accounted for by the model) and common variance (β = 0.18) with the three other predictors, and in particular with the Orthographic Decision test (see last column of Table 4). Overall, the Computation Strategies test contributes appreciably to the explained variance of the original model (*R*^2^ = 42.6%). Note that the contribution of the Computation Strategies test to the reading fluency model is a different finding from the above reported contributions of the Orthographic Decision and Arithmetic Facts tests, because it explains additional variance to that explained by the original model; thus, this predictor accounts for variance that is not captured by any of the predictors in the original reading model. Possible interpretations of this unexpected finding are presented in the section “DISCUSSION.”

When the Computation Strategies test is added to the model of spelling accuracy (see Table 4), its contribution is negligible in terms of unique variance (β = 0.004, 1.2% of the total variance explained) but more substantial in terms of shared variance (β = 0.08), most of which with the Orthographic Decision and Single Pseudo-word Repetition tests (see last column of Table 4). Overall, the Computation Strategies test contributes to the explained variance of the model with *R*^2^ = 26.9%.

### Repetition of Pseudo-Word Series Test

The Repetition of Pseudo-word Series test plays a moderate contribution to reading fluency and calculation accuracy (see Table 3). Thus, it accounts for 8.5% of the unique variance in the case of reading (fluency) with a β of.03 and 14.0% of the unique variance in the case of calculation (accuracy) with a β of.03. In both analyses, it also contributes in these models in terms of shared variance. By contrast, it does not appreciably contribute to the calculation speed model.

When the Repetition of Pseudo-word Series test was added to the original models (Table 4), this predictor plays a marginal unique contribution in all the analyses. However, it shares a large quote of variance with predictors in the model of Reading speed (β = 0.17), in particular with the Orthographic Decision test. It also shares variance (β = 0.10) with predictors of calculation accuracy (in this case the shared variance is with all the predictors in the model; see last column of Table 4), while its shared contribution with the predictors of calculation speed is limited (β = 0.03). The overall contribution of the Repetition of Pseudo-word Series test is moderate in the case of reading fluency (*R*^2^ = 36.8%) and calculation accuracy (*R*^2^ = 36.6%) and minimal in the case of calculation speed (*R*^2^ = 8.9%).

### Orthographic Decoding: Visual-Auditory Pseudo-Word Matching Test

Finally, there are predictors that exert an influence mainly (or only) on a specific behavior. Performance in the Visual-auditory Pseudo-word Matching test is a predictor of reading fluency, but does not appreciably account for unique variance in the case of calculation speed (β = 0, 1.3% of the total variance explained by the model) or calculation accuracy (β = 0.02, 9.9% of the total variance explained by the model) and also contributes little, although not zero, to common variance (see Table 3). Interestingly, the Visual-auditory Pseudo-word Matching test also does not contribute to the prediction of spelling either in terms of unique variance (β = 0.01, 2.5% of the total variance explained by the model) or shared variance (see Table 3).

When the performance in this test is added to the original reading and calculation models (Table 4), it does not contribute in terms of unique variance (β = 0 in all models) but only in terms of shared variance and only for calculation speed (β = 0.08) and accuracy (β = 0.07). In particular, this task shares variance with the Arithmetic Facts test (see last column of Table 4). Overall, the contribution of the Visual-auditory Pseudo-word Matching test in these analyses is limited (ranging *R*^2^ = 2.2 to 27.0%).

### Number Order Test

A partially similar pattern is present in the case of the Number Order test. While it contributes to the models of calculation, its predictive power in the case of reading and spelling is limited. In the case of reading, it contributes little in terms of unique variance (β = 0, 0.5% of the total variance explained by the model) as well as shared variance (see Table 3). In the case of spelling, it moderately contributes in terms of unique variance (β = 0.05, 27.8% of the total variance explained by the model which is, however, rather low).

As reported in Table 4, when this task is added to the predictors of reading, the explained variance of the model does not increase: in fact, the unique contribution is null and the shared variance is moderate (0.06) and without a detectable pattern of association with other tasks. The overall contribution of the Number Order test in this analysis is limited (*R*^2^ = 12.1%). In the case of the original spelling model, the explained variance passes from 29.2% to 30.8% due to the moderate unique contribution of this task to the model (β = 0.02) and the large quote of shared variance (β = 0.12; 39% of the overall variance) shared especially with the Orthographic Decision test and with the Orthographic Decision jointly with the Pseudo-word Repetition test. The overall contribution of the Number Order test in this analysis is moderate (*R*^2^ = 43.8%).

### Single Pseudo-Word Repetition Test

As reported in Table 3, the Single Pseudo-word Repetition test contributes to the model of spelling but does not appreciably account for unique variance in the case of reading (β = 0.01, 1.5% of the total variance explained by the model). It also does not contribute much unique variance to calculation accuracy (β = 0, 1% of the total variance explained by the model), while it moderately contributes to calculation speed (β = 0.01, 7.9% of the total variance explained by the model).

Finally, when performance on the Single Pseudo-word Repetition test is added to the other models (Table 4), in each case the unique variance is nil and the shared variance is moderate. Overall, the Single Pseudo-word Repetition test contribution in these analyses is limited (ranging *R*^2^ = 5.9 to 16.4%). Thus, by and large this ability predicts only the spelling behavior.

## Discussion

The Discussion is organized in three parts: (A) first, we illustrate and comment the results of the present analyses; (B) then we exploit a theoretical proposal to frame our results; and (C) we present a novel model of the association between learning skills as a first step in the development of a model of comorbidity of learning disorders.

### A. Interpreting Results of Cross-Predictor Analyses

The pattern of results for the cross-over analyses would be indeed surprising from the standpoint of putatively specific learning disturbances. Several, though not all, predictors show strong influences not only on their target behavior but also on putatively non-target behaviors. Below we illustrate possible interpretations of some of these relationships. Clearly, this is a data-driven process but one that may have the potential of understanding the breadth of the influence of factors more than in the typical case in which a given factor is tested only within a single, specific domain. Note that no attempt is made to yield an entirely exhaustive interpretation of every single factor (in all possible combinations) across all behaviors. The aim is rather that of using these results for their potential heuristic role in generating hypotheses on the association of learning skills and eventually on the co-morbidity of disorders of learning behaviors.

#### Memory Retrieval and the Ability to Automatize Instances

A particularly striking pattern is that pointing to an association between the Orthographic Decision and the Arithmetic Facts tests. Both tests are “strong” predictors of a target behavior (reading/spelling and calculation, respectively), but also of the other non-target behaviors (calculation and reading/spelling, respectively). What could be the reason for this pattern?

In spite of their surface characteristics, it should be noted that the Orthographic Decision and the Arithmetic Facts tests share the requirement of calling a specific trace from memory. In the case of maths, children first learn to make computations by applying an algorithm; then, by repetitive exposure to the solution of a given simple operation (such as ”3 times 8”), they learn to directly access the solution of the operation (i.e., 24) without application of the algorithm. The Arithmetic Facts test measures this latter ability. In a similar vein, at least in a regular orthography such as Italian, children first learn to apply grapheme-to-phoneme (and phoneme-to grapheme) conversion rules to read (and spell) words. Through repetitive exposure to print, they slowly learn to directly access the target word (i.e., reading by “sight”) without passing for the conversion of graphemes into phonemes (e.g., Marinelli et al., 2015, 2016b,2020a). Within the dual route tradition, it is generally believed that access to the orthographic input lexicon facilitates reading and spelling of all words and ensure reading speed; however, this effect is clearest in the case in which the word cannot be read and spell through the sub-lexical conversion routine, as is the case of irregular words. The Orthographic Decision test ensures that the reader uses the orthographic lexicon also in a consistent orthography, such as Italian, by requiring the child to judge the orthographic correctness of a pseudo-word homophone to a real (inconsistent) word, a task that can be solved only through reliance on acquired orthographic representations not on sub-lexical mapping. Then, both in solving arithmetical facts and in carrying out orthographic decisions on inconsistent words, with increasing experience and practice children progressively pass from the application of an effortful and serial algorithm to a less demanding process based on the fast and automatic retrieval of a memory trace. Thus, the Orthographic Decision and the Arithmetic Facts tests share the requirement *to retrieve a trace in memory*, not to apply a specific algorithm.

A theory that formalizes the ability to retrieve quickly and automatically a specific memory trace is the “*Instance theory of automaticity*” proposed by Logan (1988, 1992). According to this learning theory, automatization is acquired through repetitive presentation of a stimulus: in this way, the “instance representation” of an individual object or event is stored in memory (“obligatory encoding”) and, the more repetitions, the more information becomes directly available (“obligatory retrieval”). In the course of learning, the individual’s responses to the item are progressively faster, the pace of learning being described by a power function (as originally proposed by Newell and Rosenbloom, 1981). This indicates that initial learning is fast and rate of improvement is progressively slower over target repetitions, although the function does not clearly reach a plateau (mathematically, the power function goes to zero at infinite). Still, the nearly asymptotic portion of the curve expresses a very fast and nearly constant performance, as typical of automated tasks characterized by “obligatory retrieval”.

Based on the present finding that the Orthographic Decision and the Arithmetic Facts tests are strong predictors of both the target and non-target behaviors, we propose that they both capture (at least in part) the degree of automatization characterizing an individual (e.g., how consolidated and easy to retrieve is the information that 3 × 8 = 24; or that QUOCO is not a correct spelling). Thus, the individual level of *ability to automatize instances* can offer a basis for this finding.

#### Using Contextual Information in Different Domains

Another test that showed crossed influences was the Computation Strategies test. This did not only explain variance in the two calculation models but actually increased the overall power of the model of reading by a substantial amount. This test accounts for a proportion of variance that is actually additional to that of all the predictors considered in the original reading model.

Several interpretations can be advanced to understand this unexpected finding. Here, we focus on only one based on an analysis of the characteristics of the Computation Strategies test. This explicitly requires the child to use the available information to solve the task instead of computing. Thus, knowing that 13 + 148 = 161 (presented on the left side of a sheet of paper) can be used to speed up an operation such as 14 + 149 = …… (presented on the right side) over and above the knowledge of arithmetic facts, calculation properties and abstract number representations. In other terms, the context provides information that can be used for the processing of the ongoing stimulus, greatly facilitating the computation task. Similarly, it seems possible that the same ability is useful in reading meaningful texts, i.e., the task used in the present study to measure reading performance.

Thus, to the extent to which the Computation Strategies test captures variance in a factor that can be defined as “use of contextual information”, this may account for its contribution to the model of reading. Indeed, it is well known that contextual information optimizes reading fluency (e.g., Perfetti et al., 1979; Stanovich and West, 1979; Becker, 1980; Simpson et al., 1989). Our original model of Reading fluency did not consider processing of contextual information, but the crossed influence of the Computation Strategies test suggests that a model of reading could be enriched by considering this fourth factor. Clearly, this is a *post hoc* interpretation but one that can be subjected to empirical test. In particular, if the above speculation is correct, one would expect that performance on the Computation Strategies test would not contribute to variance in reading lists of unrelated words (a test not included in the present study).

#### Role of the Ability to Integrate Task Subcomponents

Some factors exerted an influence that was selective for a specific parameter across behaviors. In particular, RAN contributed in explaining variance to both reading fluency and calculation speed but did not contribute in explaining variance across measures of accuracy (both in the case of spelling and calculation). The RAN task requires the child to integrate the processing necessary for selecting the landing point of the next fixation with processing of the actual target and identification, as well as access the name of the visual object, its maintenance into short-term memory and actual utterance. These activities have to be effectively synchronized for allowing a fluent performance across a matrix containing several different targets. When seen in the light of reading, this skill appears to mark a dimension of “integration of reading sub-components”; indeed, RAN requires all the operations typical of text reading, apart from orthographic analysis (Protopapas et al., 2013; Zoccolotti et al., 2014). This interpretation is supported by the evidence which shows that the relationship of RAN tasks to reading is diluted if the number of alternative targets is reduced and the subject has to produce a single repetitive response (Georgiou et al., 2013) or a single, discrete presentation of RAN-type stimuli (instead of multiple) is used (Georgiou et al., 2013; Zoccolotti et al., 2013). In trying to account for the influence of RAN in the case of the speed of performance in mental calculations, one may refer to a similar interpretation. Indeed, the subject has to integrate processing necessary for selecting the landing point of the next fixation with processing of the actually fixated information, as well as its maintenance into short-term memory in order to apply the required processing (sum, subtraction, etc.). Thus, one can think that RAN performance marks a cognitive dimension, which is present in both reading and calculation, concerning the “ability to integrate task sub-components” in order to achieve a fluent performance.

#### Predictors Specific for Single Behaviors

Finally, some variables exerted an effect that was quite specific for a single behavior. Thus, the Visual-auditory Pseudo-word Matching test was predictive of reading skill but not (or minimally) for spelling and calculation. The Single Pseudo-word Repetition only entered in the prediction for spelling and not in any non-target cross-over model. Finally, the Number Order test (which marks the cognitive dimension of “Symbolic number representation”) had an influence almost only in the case of the target behavior, i.e., calculation skills. It seems that these tasks tap processes that are specific for a single behavior.

### B. A Multi-Level Approach to Co-morbidity in Learning Disturbances

Overall, the results of the cross-predictor analyses indicate that some predictors have a general influence across various behaviors while others predict different behaviors but only for a specific parameter, such as fluency but not accuracy, and still others are specific for a single behavior. These findings cannot be easily fit into a framework considering a single level of explanation. Rather, it appears that predictors act at different levels of generality and such characteristic should be kept into account in trying to propose a comprehensive interpretation of association of learning skills. This in turn might help to better understand the co-morbidities across different learning disabilities.

As indicated in the Introduction, one traditional distinction is between “competence” and “performance,” originally put forward by Chomsky (1966) in the discussion about language. In general, competence is referred to as the abstract, general capacity to process in a given domain (such as language in the case of Chomsky). The concept of “performance” refers to the fact that what we measure in a given individual with a given task is not a direct measure of his/her competence in the domain, but the result of an interaction between competence and the specific characteristics of the task. So, in a sense, the critical difference between competence and performance is that the former is task-independent while the latter is task-specific. In this perspective, all measures of a given behavior depend upon both the competence in a specific domain and the performance on the specific task.

The value of making such a distinction is that one may assume that deficits in a specific competence (e.g., reading) will show up pervasively across different types of tasks in the domain (such as reading meaningful texts, list of words, or pseudo-words, either printed or flashed alone on a computer screen, or presented by rapid serial visual presentation, etc.). Conversely, other defects may appear contingently to the requirements of the actual task (for instance, a child may be below the norm in reading a text but not in reading single short words; may have spelling problems under dictation but be fair in writing his/her own ideas; may have problems in maths under time pressure while being accurate if enough time is given). In all these cases “performance” components are on the foreground.

The importance of such “performance” or “processing” deficits should not be overlooked (for a discussion see Bishop, 1997). In real life, we read or do calculations under specific conditions, which need to be duly fulfilled for an effective outcome. Much the same occurs in a clinical setting where reading, spelling or maths deficits are typically investigated largely using standardized tasks similar to the typical conditions of stimulus presentation that children face during their school experience (and that are typically graded according to the number of years of school experience). It should also be kept in mind that any measure of reading, spelling or maths behaviors will depend upon both competence and performance and separating these two components is inherently difficult although it may be attempted by the use of *ad hoc* analyses.

Further, we propose that a third level of explanation should be added to fully account for the complexity of results and is related to the process of “learning” or “acquisition”, and particularly to its automatization phase. By and large, acquisition occurs through the effect of practice. First of all, extended practice is critical to produce automatized responses to specific target items. This would contribute to the ability to read (or spell) words (or make multiplications) not based on grapheme to phoneme conversion (or counting digits), but on direct memory retrieval of specific target items (or “instances”; Logan, 1988, 1992). Thus, through extended practice the child learns specific items (e.g., regular frequent words, such as “house,” or irregular words such as “pint,” or the output of simple mathematical operations such as 3 × 8 = 24 or 4 + 2 = 6). In keeping with Logan (1988, 1992) proposal, learning specific instances directly contributes to the automatization, and obligatoriness, of responses, contributing to make reading, spelling and doing maths fast and smooth processes. Learning disabilities do not refer to the complete inability of the child to learn to read, spell or to do calculations as much as *the inability to do so smoothly and efficiently*. Thus, for example children with dyslexia characteristically read in an effortful, not automatic fashion; in order to read, the child has to place all his cognitive resources on decoding the text with little residual ability left for comprehension. Thus, we propose that also the *ability to learn specific instances* should be included in a three-level framework of interpretation in explaining the acquisition of learning skills.

However, practice influences all processes of learning a skill, such as reading, spelling or maths, including the acquisition of competence and the tuning of performance skills. For example, in the case of reading, through extended practice, the child has sufficient experience with the process of converting graphemes into phonemes in a given orthography, which may be the condition to activate and form a specific reading “competence” (see further comments below). Through extended practice, the child also optimizes his/her capacity to read under the typical task format used in school (e.g., Girelli et al., 2017). Thus, in several languages, words are presented horizontally, printed in black on a white surface and the child learns to read them in a left-to-right direction, slowly acquiring the capacity to smoothly read sequences of words in a text (not only isolated targets). Thus, practice favors the emergence of a reading “competence” as well as optimizes efficiency in specific task conditions (“performance”).

Some general functions and characteristics of the competence, acquisition and performance levels are summarized in Table 5.

**TABLE 5:** Main functions and characteristics of competence, acquisition and performance levels as related to individual differences in learning skills and comorbidity of learning disorders.

	Function(s)	Characteristics	Specificity/overlap
Competence	Ability to activate a specific set of representations and processes	- Domain-dependent - Task-independent - Sensitive to practice	Dissociation of deficit may be present to the extent in which different processes rest upon different sets of representations and algorithms

Acquisition	- learning specific rules and/or regularities (*algorithms and core competence*) - learning direct memory traces (*instances*) which are automatically retrieved and coercively activated in the presence of appropriate stimulation - Upon practice, the child gets accustomed to the *typical task format*, characteristic of a given behavior (e.g., reading a text in a left to right manner).	- Domain-dependent - item specific - Domain-independent It follows a general law a practice, characterized by a slow pacing of learning (long periods for over-learning and automatic responding) - Partially domain-dependent	Consolidation of instances may dissociate from learning of algorithms. Deficits in developing automaticity may lead to learning disorders across different domains (comorbidity)

Performance	Actual performance depends on the characteristics of the task which may call into action different processes depending upon the specific competence involved or/and the characteristics of the task itself (e.g., a speed task).	- Task-dependent - Partially domain-dependent - Sensitive to practice	It may lead to both associations and dissociations of learning skills (and disabilities) depending on the degree of overlap of: task-specific processes and their interaction with specific “competence” requirements

### C. A Model of Learning Skills Based on Competence, Performance and Acquisition (Automatization) Levels

**FIGURE 2:**
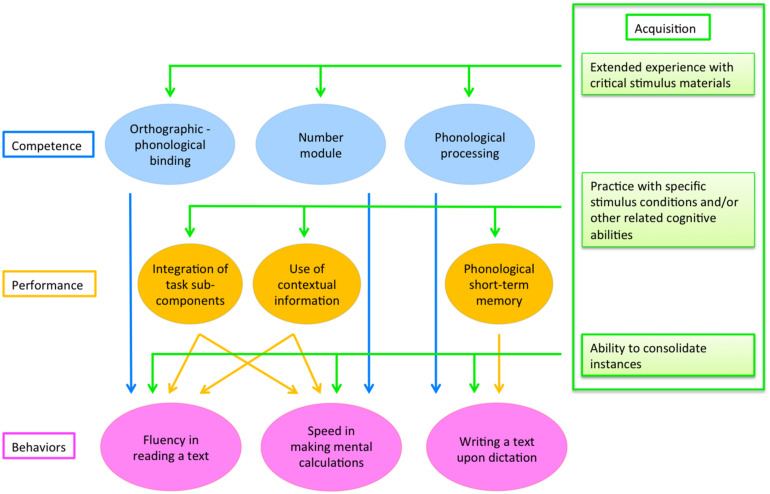
A multi-level model of learning cognitive skills. Target behaviors are expressed in terms of task-specific exemplars. As for mathematical skills, only the case of calculation speed is shown. A description of the figure is presented in the text.

Drawing on the distinction among competence, acquisition and performance levels it is possible to outline a unitary, multi-level model of reading, spelling and maths skills. The model is illustrated in Figure 2. For the sake of presentation, only some of the factors possibly affecting performance are indicated; furthermore, for maths skills, only the case of calculation speed (but not accuracy) is shown. Note that the architecture of this model is considerably more general than that presented in Figure 1; however, also this can still be considered as a proximal model to the extent in which it envisages explicit relationships (depicted by arrows in the figure, and made explicit in the text) between predictors and different behaviors.

The model illustrates the possible sources of associations and dissociations among reading, spelling and maths skills. In particular, it is assumed that independent competences (represented in blue in Figure 2) are present and specific for these three behaviors and that this may account for dissociations among learning skills (as well as disorders). On the other hand, association among learning skills may be due to an acquisition factor (green lines coming from the three acquisition boxes represented in Figure 2), i.e., the “ability to consolidate instances” which is responsible for automatized responses in reading, spelling and maths.

#### Dissociations Among Learning Behaviors

The view that specific, different competences underlie the three behaviors considered is supported by the literature, although the same literature is rich of possible alternatives on the nature of the competences involved in reading, spelling and maths.

##### Reading competence

In the case of reading, one line of research emphasized the role of phonological processing (e.g., Stanovich, 1988). However, a systematic theory-based test of this hypothesis indicated that both English and Hebrew individuals with dyslexia showed the expected sensitivity to general phonological contrasts, although also had moderate deficits in some (though not all) phonetic categories (Berent et al., 2013, 2016). The authors concluded that individuals with dyslexia show spared phonological competence while they may be impaired in some phonetic tasks (i.e., pointing to deficits in “performance” processes). Similarly, also Ramus and Szenkovits (2008) reported spared phonological competence with deficits in children with dyslexia associated to specific task conditions (i.e., “performance” factors).

In previous studies by our research group, we focused on a different alternative interpretation, i.e., that reading competence expresses the ability to form a pre-lexical representation of the orthographic string. To test this possibility, we examined performance of children with dyslexia across several different experimental conditions to cancel out the effect of performance and let emerge the non-task specific characteristics of the deficit. In a series of investigations, we adopted this approach by applying models of global performance, such as the rate and amount model (RAM; Faust et al., 1999) or the difference engine model (DEM; Myerson et al., 2003) to study reading deficits. Results indicated that the same “global” defect was present whenever a string of letters (not a single letter or bigram) was presented (De Luca et al., 2010), and whether or not it constituted a word (i.e., the same deficit was observed in the case of words, pseudo-words as well as unpronounceable non-words; Marinelli et al., 2014). Importantly, the difficulty of children with dyslexia did not extend to non-orthographic materials (such as pictures; Zoccolotti et al., 2008; De Luca et al., 2017) or responding to stimuli presented in a non-visual modality (i.e., acoustically; Marinelli et al., 2011). These results appear consistent with the idea that the basic competence involved in reading is the ability to form a pre-lexical representation of the orthographic string (also called “graphemic description” by Marsh and Hillis, 2005).

This view is consistent with a comprehensive model of the putative “competence” of reading that has been the focus of a large series of neuroimaging investigations by Dehaene and colleagues (e.g., Dehaene et al., 2005). In summarizing their studies, Dehaene et al. (2005) have proposed a local combination detectors (LCD) model. Interestingly, according to Dehaene and Cohen (2007), tuning of the VWFA represents an instance of cultural re-cycling, such that, upon appropriate exposure to a given orthography, neurons in the areas devoted to visual object recognition optimize their responses to specific stimuli, such as letter strings (bigrams, trigrams and quadrigrams). Note that, in this view, the reading competence is associated to the ability to efficiently read letter strings which are represented within the most frequent words in a given language, not necessarily the capacity to read specific words. While the LCD model by Dehaene et al. (2005) emphasizes visual processes, in several parts of their formulation they also indicate that this sensitivity must be coupled with specific phonological activation. This point has been made most cogently by Blomert (2011) who summarized a number of imaging studies pointing to the presence of specific orthographic-phonological connections (Blau et al., 2008, 2009, 2010) and refers to this pattern in terms of orthographic-phonological binding. Thus, extended practice with orthographic materials is necessary to reach a fine tuning of visual mechanisms and strong connections with language areas; the first years of schooling are crucial to this aim (Blomert, 2011) but there are children who show difficulties at these early stages, indicating a selective difficulty in the acquisition of the core reading competence.

The challenge to define the core competence that characterizes the process of reading is still open. However, based on the above quoted evidence, we propose as a working hypothesis that the key competence in reading refers to the ability to form and activate pre-lexical processes of “orthographic-phonological binding” upon the presentation of orthographic strings. Orthographic-phonological binding is represented in Figure 1 in the oval blue. Notably, the results of the present study are well in line with this proposal. Thus, the Visual-auditory Pseudo-word Matching test entered in the prediction of reading but not in that of maths or spelling.

##### Maths competence

Literature on numerical skills clearly indicates a separate key competence than reading. In particular, several authors have proposed that the core competence regards the *ability to represent and process numerosity* (Landerl et al., 2004; Butterworth, 2005; Wilson and Dehaene, 2007). Butterworth (2005) explicitly refers to the need for this skill to be tuned through adequate exposure. In the present experiment, a measure which can putatively capture this skill is the performance in the Number Order test. Consistently, performance in this test entered in the model of numerical skills and explained an important portion of variance, but not in that of reading or spelling.

Note that authors differ in their consideration about whether this skill should be seen as general or two distinct representational systems for symbolic and non-symbolic numerosity tasks should be envisaged (e.g., Butterworth, 2005; Sasanguie et al., 2014). Here, partly because of the complexities inherent in carrying out non-symbolic numerosity tasks, we only focused on symbolic tasks. So, present data are not informative concerning this distinction and further work seems necessary.

Recently, Moll et al. (2019) reported that co-morbidity between reading and maths disorders depends upon the maths subskills considered. Thus, there was a stronger association between literacy and arithmetic than between literacy and magnitude processing (measured both as comparison among digits and perception of dots numerosity). This finding is in line with the present proposal that the core competence of representing and processing numerosity (Number module in Figure 2) accounts for the specificity of the maths disorder, not for its co-morbidity with other learning disabilities.

##### Spelling competence

It is somewhat more complex to adjudicate whether reading and spelling rest on the same or different competences. As stated above, reading competence heavily rests on the ability (made possible by specific areas in the left temporal-occipital cortex) to activate visual traces of letter strings such as bigrams, trigrams and quadrigrams. On the converse, it is generally believed that spelling closely rests on the *availability of well-defined phonological traces* (e.g., Perfetti, 1992). Therefore, we focus on such ability as the specific competence supporting spelling (see the blue oval in Figure 2).

The results in our previous report (Zoccolotti et al., 2020) and the present analyses are consistent with this view. While phonological markers did not appreciably contribute to the prediction of reading, they did so in the case of spelling. Notably, two phonological tests (Single Pseudo-word Repetition and the Repetition of Pseudo-word Series) contributed to the best model for spelling accuracy and they did so in a suppressive interaction from each other (Zoccolotti et al., 2020). The performance in the Repetition of pseudo-word series seems closely coupled to the specific requirements of the task. Thus, in the “Nonna Concetta” test, dictation stresses the ability of the child to maintain in short term memory a complex sequence of phonological information. Accordingly, one may propose that the Repetition of Pseudo-word Series test captures variance associated to the specific task characteristics (i.e., performance). By contrast, one may envisage that the variance of the Single Pseudo-word Repetition test may be more directly related to the core phonological competence of spelling. If this hypothesis is correct, this latter test should enter in the prediction of spelling skills independent of the specific task, i.e., as a marker of the competence in spelling.

In Figure 2, we tentatively point to the three above defined competences in terms of independent processing (as sketched by the three separate blue arrows pointing to the three behaviors). Core competence factors largely account for the presence of distinct components of variance in reading, spelling or maths skills and potentially for the dissociability of deficits in these learning domains. Yet, a note of caution is in order on this conclusion. In fact, it should be kept in mind that competence cannot be directly probed with a single task; thus, a thorough test of a given competence requires direct control of the role of task requirements, which in turn would require *ad hoc* investigations.

#### Associations Among Learning Behaviors

By contrast, associations (or co-morbidity) are mostly explained by the presence of a domain-independent factor (“ability to consolidate instances”). The direct effect of this factor on behaviors is indicated in Figure 2 by the horizontal green line from the “Ability to consolidate instances” box pointing with three green arrows to the three target behaviors. Accordingly, individual skill in automatizing would span over reading as well as spelling and maths. We previously referred to the distinction between proximal and distal factors. In describing distal factors, frequent examples in the literature refer to domain general processes, such as short-term memory or attention. In the view proposed here, the ability to automatize is seen as domain general process but one for which an explicit relationship of its influence over the dependent measures is envisaged, i.e., it is described in proximal terms.

In particular, the ability to automatize is a factor that contributes to efficient performance. Conversely, poor ability in forming instances does not make the behavior impossible but rather has the more specific effect of preventing fast and fluid reading, efficient spelling and fast and efficient calculation. As stated above, children (and even more so adults) with dyslexia are not unable to read but their reading is cumbersome, inefficient and ultimately tiring, characteristics that indicate a controlled, voluntary mode of processing (Schneider and Chein, 2003). This contrasts with the smooth and efficient decoding of the typically developing peers, which marks their pre-attentive, automatic processing (Schneider and Chein, 2003). Thus, lack of automaticity does not necessarily indicate an impaired core reading competence (which of course may also be present), but would indicate a deficit in a component necessary for fluent reading, and one that can be a source of at least partial associations.

Thus, following our hypothesis, a lack of automaticity in reading should be associated with a deficit at maths level, in the form of difficulty to retrieve arithmetic facts. Indeed, adults with dyslexia were found defective in their ability to retrieve arithmetic facts, although their numerical representations were spared (De Smedt and Boets, 2010). Accordingly, they show an “incomplete” pattern of co-morbidity (meaning that the association is not between behaviors as such, but between sub-components of different behaviors). In this vein, one may hypothesize that other forms of incomplete co-morbidities may be present (for a discussion on this point see Moll et al., 2019). For example, children with dyscalculia including a limited capacity to retrieve arithmetic facts should show selective deficits in reading irregular words or choose among homophonic versions of orthographic strings with inconsistent transcription, even in cases in which there are no sufficient elements for a formal diagnosis of dyslexia. Further research is needed to confirm this hypothesis.

Another prediction that can be advanced is that deficits that can be ascribed to a defective ability to consolidate instances should emerge more clearly late in the course of development, when typically developing children have consolidated their knowledge of many items allowing fast and smooth reading. Findings along this line have been advanced in terms of spelling skills by Angelelli et al. (2010b). They noticed that children with dyslexia showed parallel deficits in spelling but the characteristics of the writing deficit changed as a function of age. In third grade, children showed a generalized deficit encompassing all stimulus categories while, in fifth grade, there was a clear prevalence of spelling errors for inconsistent words, i.e., words which require the retrieval from memory of the lexical representation. In a parallel study, Angelelli et al. (2010a) examined the consistency of this lexical deficit between a reading (orthographic decision) and a spelling task. Fifth grade children with dyslexia showed a parallel impairment in both tasks and, in particular, showed item consistency across reading and spelling, i.e., they were impaired in judging the orthographic correctness of the very same words with irregular transcription which they failed to spell. Thus, their lexical deficit was item specific but consistent across reading and spelling, a pattern consistent with the idea of a cross-modal defect in consolidating specific instances. Finally, Marinelli et al. (2017) recently reported that, in spite of their item-based lexical deficit in both tasks, children with dyslexia showed appropriate sensitivity to the distributional information of sound-spelling mappings at sub-lexical level and such knowledge facilitated both spelling and reading, allowing for partial compensation of their lexical deficit.

Overall, it is proposed that the putative lexical deficit shown by children with dyslexia in both reading and spelling can be ascribed to a more general defect in consolidating individual instances. Consistently with the “Instance theory of automaticity” (Logan, 1988, 1992), such deficit (a) emerges more clearly late in the course of development, when automatization is acquired in most typically developing children (Angelelli et al., 2010b; Marinelli et al., 2017) and (b) is characterized by item specificity (Angelelli et al., 2010a; Marinelli et al., 2017). Furthermore, in keeping with the idea that a deficit in the ability to consolidate instances is domain-independent, the deficit is quite consistent between reading and spelling (Angelelli et al., 2010a). Finally, the deficit is independent from a deficit in competence as such (Marinelli et al., 2017). So, these data are consistent with the idea that some children may suffer from an acquisition defect which is particularly evident in reading and spelling tasks calling for item specific knowledge of words with inconsistent mapping. Based on the present hypothesis, we would expect these children to be also selectively impaired in arithmetic fact retrieval, a prediction that can be the object of future investigations.

#### Previous Studies on Automatization Deficits in Dyslexia and Difficulties Inherent to the Evaluation of This Hypothesis

The idea that the lack of automaticity may be a cause of dyslexia as well as other learning disturbances has already been advanced in the literature (Nicolson and Fawcett, 1990). Interestingly, these authors do not propose a deficit in automaticity as a single cause of dyslexia but rather they envisage an additional role for this factor, one that, however, may work for different learning disabilities, thus “*reuniting the developmental disorders*“ (Nicolson and Fawcett, 2007). The position we take here is similar in this respect. However, a key distinction to the proposal by Nicolson and Fawcett is that we take a proximal approach and propose that the ability to consolidate instances is part of the multi-level model accounting for reading, spelling and doing maths. By contrast, in their original formulation, Nicolson and Fawcett (1990) rested on a distal approach. This led to experiments testing whether children with dyslexia were impaired in cerebellar-like tasks, such as balancing a rod or maintaining a posture. Perhaps, such relationships do exist, although substantial failures to replicate the original findings have been put forward (e.g., Wimmer et al., 1999; Ramus et al., 2003). At any rate, we propose that performance on these tasks only capture a long-distance relationship (in a distal perspective), while a much more specific formulation is possible within a proximal perspective. In particular, the model developed here allows specifying what should be predicted by the automaticity component, i.e., the ability to foster performance by activating direct responses to single, well-practiced items, an ability which is domain general because it holds across different learning tasks. Thus, no direct relationship is expected with reading or maths competence but with the ability to activate *specific reading or spelling traces or arithmetic facts*.

Research on the relationship between automaticity and reading illustrates a key problem in obtaining a measure of the individual sensitivity to acquire fast responses to specific events with practice, as adjudicated by the “Instance theory of automaticity” of Logan (1988, 1992). This essentially makes predictions over relatively long periods of training. In this vein, it is difficult to find a single task that directly measures individual differences in automaticity. By definition, tasks at “just one point in time” are sensitive to the product of a process that, however, depends upon both the initial performance on the task, the rate of improvement and the degree of practice. Thus, to obtain a process measure of automaticity, it is necessary to examine the course of acquisition, not only the performance at one point in time. The model of Logan (1988, 1992) provides clear predictions of how to measure performance to the extent in which it specifically envisages a power function rate of learning for new tasks; this can be expressed at both group and individual basis in terms of the coefficient of the curve as well as its setting parameters.

Nicolson and Fawcett (2000) were aware of this complexity and carried out one of the few studies based on long-term learning in relationship to dyslexia. They investigated the effect of a long-term training on a keyboard spatial task and a choice reaction task and reported a greater difference between the initial and final performance in typically developing children than in children with dyslexia, though the rate of learning was not different in the two groups. In the perspective advanced here, it is difficult to directly extrapolate from this type of tasks (which were conceived in a distal perspective) the performance in learning tasks, such as reading, spelling and doing maths. At any rate, this study is one of the first attempts to examine rate of learning in children with learning disorders over a relatively large time-scale. Other more recent studies (Martens and de Jong, 2008; Pontillo et al., 2014; Suárez-Coalla et al., 2014; Kwok and Ellis, 2015) have focused on reading tasks and examined the ability to improve performance on individual orthographic items devoid of meaning (pseudo-words) upon repeated presentations. In general, these studies indicate that rate of learning is slower in children with dyslexia. Characteristically, with training typically developing children considerably reduce their sensitivity to pseudo-word length while children with dyslexia do not (or do so to a much lesser extent). These findings with originally unknown items (pseudo-words) are in keeping with the idea that children with dyslexia have a deficit in optimizing their performance to individual items with training. Thus, at least part of the reading deficit may be ascribed to a reduced tendency to build and automatize responses to individual items.

#### Role of Task Requirements (Performance Factors) in Co-morbidity

Other sources of association among behaviors (and henceforth of co-morbidity) may originate from similarities among task requirements across different behaviors (see performance labels in the yellow ovals and arrows in Figure 2). As stated above, reading in standard conditions is a task with an inherent speed component. Similarly, in order to achieve an effective skill in making the moderately complex calculations typical of high-school teaching, adolescents have to learn to quickly and strategically activate algorithms and arithmetic facts knowledge. A task like RAN, which is particularly sensitive to the “*integration of task sub-components*” may well capture the “fluency” variance associated with performance in these two behaviors (as sketched by the two yellow arrows from the box pointing toward both reading and maths behaviors), possibly contributing to their frequent association. Consistently, a recent meta-analysis of 38 studies on the relationship between RAN and maths found that the correlation between RAN and fluency in doing calculations (speed) was on average higher than that between RAN and maths accuracy (Koponen et al., 2017).

In this vein, note that, presumably due to the diluting effect produced by the requirement of sequentially manually articulating the script (i.e., a relatively slow procedure), spelling does not usually pose such stringent time constraints in the integration of task sub-components (and spelling speed is generally not taken as a critical measure of orthographic competence). Consistently, performance on RAN tasks did not predict spelling performance, a finding in keeping with previous research in the literature (Moll and Landerl, 2009; Furnes and Samuelsson, 2011). Conversely, depending on the type of performance (spontaneous, from dictation etc.) writing may more or less call into action *phonological short-term memory* processes (as sketched by the arrow pointing only to spelling behavior).

Recent evidence on the partial dissociability between reading and spelling deficits also points to the mediating role of the speed component in reading (Mehlhase et al., 2019). Thus, spelling deficits were associated to deficient storing of orthographic representations in long-term memory while isolated reading fluency deficits were associated to spared orthographic representations but slowed access to these representations.

A full account of the role of “performance” factors is difficult as, by definition, they are task-dependent and one can use a large variety of tasks to study reading, spelling or doing maths. However, we feel that this is not a sufficient justification to exclude performance factors from a model of co-morbidity. Indeed, as emphasized by Pennington (2006), co-morbidity occurs among “complex” behaviors and the contribution of performance factors is indispensable if such behaviors need to be accounted for. In this vein, note that cognitive models of reading, spelling and maths give little, if any, space to performance processes (for a discussion see also Bishop, 1997). For example, models, such as the DRC, the CDP + or the triangle model, do not take into consideration the time constraints typical of the reading tasks and limit their formulation to an abstract analysis of single word reading. It is well established that reading deficits occur already at the level of single word processing, and predicting single word reading may be instrumental to build a model of the reading competence. However, experiments also show that the requirement to read multiple stimuli, such as in functional reading, selectively aggravates the reading deficit (e.g., Zoccolotti et al., 2013), a finding that has no clear space in current reading models. Thus, simulations based on models such as DRC or CDP + account for some effects shown in the literature (such as frequency, lexicality, regularity by frequency and so on) but provide a limited prediction of reading performance in everyday conditions. This limitation does not only affect our understanding of the target behavior (e.g., reading) but it also dampens our understanding of the sources of the co-morbidity among complex behaviors as they may not depend only upon the pervasive role of automatization deficits but also on complex interactions between competence and performance factors.

By contrast, some limited consideration of the actual need to implement an actual behavior is present in models of spelling (e.g., Hillis and Rapp, 2004). Thus, dual route models envisage that graphemes have to be converted into letter shapes for actual, motor performance. Yet, this terminal process is entirely encapsulated and supposedly does not interact with the central spelling processes. Interestingly, empirical evidence seems to go in the opposite direction of this independence. For example, it was reported that after training in handwriting, children showed significant improvements in compositional fluency (Graham et al., 2000). In a similar vein, Jones and Christensen (1999) reported that orthographic-motor integration accounted for a large proportion of variance in written expression over and above the effect of reading. These findings are in keeping with the idea that performance factors interact in complex ways with competence to determine actual behavior.

## Conclusion, Limits and Perspectives

The general aim of the present study was to develop a proximal model of the factors accounting for individual performances in learning skills, which could as well provide a base for understanding the co-morbidities among learning disorders. Our focus on proximal predictors, i.e. featuring explicit relationships between predictors and behavior, is in recognition of the great difficulty to pinpoint distal relationships. Present results indicated considerable overlap between the predictors of reading, spelling and maths. This overlap cannot be simply interpreted in terms of general cognitive abilities, as measured by well-established cognitive tests, because these latter predicted only a limited amount of variance. Notably, proximal predictors of reading accounted for performance in calculation tasks considerably better than did control factors measuring general cognitive abilities and much the same occurred when other crossed analyses were carried out, such as predicting reading with maths predictors and so on.

To interpret these results, we propose that it is necessary to separately consider factors in terms of competence, performance as well as acquisition/automatization. This multi-level distinction seems particularly suitable to account for the association of learning skills and, in perspective, for the widespread presence of co-morbidity among learning disorders. We have tentatively proposed that the three skills (reading, spelling and calculation) are made possible by the development through extended practice of three different abstract competences; this separation may account for the presence of partial dissociations among learning disorders. By contrast, crossed predictors point to associations (or co-morbidities). In particular, these can be seen as due to the domain general influence of the ability to automatize responses to specific items. Furthermore, also overlap between task characteristics (such as time pressure or the use of contextual information in different domains) may contribute as performance factors in producing associations between learning behaviors.

Three main conclusions of the present study come to the foreground.

Firstly, a proximal approach such as that presented in Figure 2 may have the potential to interpret co-morbidities; in particular, it provides the ground to predict both associations and dissociations among disorders. This is an important advancement over previous models of learning focusing on a single behavior (or deficit) which, by definition, left associations with other learning disorders aside (for a discussion see Pennington, 2006). A proximal approach is potentially able to focus on a limited set of possible dimensions underlying each behavior (as it was done in Zoccolotti et al., 2020) and forces to interpret also unexpected relationships (as in cross-over analyses in the present study) in terms of explicit links between predictors and behavior. Indeed, the present results indicate that seeing learning disorders in their multi-level complexity may actually help in better interpreting every single disturbance to the extent in which it provides a framework to interpret cross-modal predictors. For example, the presence of deficits in developing automatic responses to individual targets (such as reading a word or retrieving arithmetical facts) may be more easily interpreted within a general learning framework than when examining a single disorder.

A second general conclusion is that a cognitive multi-level approach that distinguishes between competence, performance and acquisition factors, may be particularly effective in framing learning disturbances (Berent et al., 2013, 2016). In fact, reading models, whether in the cognitive or connessionist tradition only focus on the description of a competence and largely ignore performance factors (Bishop, 1997). However, both competence and performance influence the actual measures taken for reading, spelling and doing maths and failing to distinguish between the two hinders the actual possibility to test such models.

It is less obvious the need to assume that acquisition should be considered as a separate level of analysis. After all, practice affects all levels of processing. Thus, in the particular case of reading, spelling and maths, prolonged practice is necessary for creating a competence, possibly by tuning the response selectivity of moderately flexible cortical areas (as envisaged in the cultural re-cycling hypothesis; Dehaene and Cohen, 2007). Similarly, practice affects performance factors, such as the ability to process multiple targets in reading (e.g., de Jong, 2011; Protopapas et al., 2013) or using contextual information in maths and reading (present data). However, in our model we propose that “the ability to consolidate instances” should be seen as a separate level of analysis.

On general grounds, it has been emphasized that the learning system has to balance the need of “*detecting regularities in the world through generalization versus encoding and remembering particular events and their details through mnemonic specificity*” (Keresztes et al., 2018). Recent evidence indicates that the developmental lag between these two general functions (the former emerging ontogenetically much earlier than the latter) is at least partially mediated by differences in the timing of hippocampal maturation (Keresztes et al., 2018). Thus, we may extend this view to learning abilities such as reading, spelling and calculation, with “competence” as a form of learning by generalization and “ability to consolidate instances” as a form of learning specific events (whether words or arithmetic facts).

Third, we think that the present proposal has the potential to drive new research. Following the seminal work of Pennington and colleagues (Willcutt et al., 2005, 2010; Pennington, 2006), there has been an increasing interest in the search for the cognitive factors accounting for the co-morbidity between disorders such as dyslexia and dyscalculia (see also Pennington and Bishop, 2009). Still, the search for the common factors accounting for the overlap among learning disorders has proven largely unconclusive (Willburger et al., 2008; Landerl et al., 2009; Slot et al., 2016; Wilson et al., 2015; Cheng et al., 2018). In the view proposed here, learning disorders emerge when the combined effect of abilities/deficits in competence, performance and acquisition levels passes a threshold of overall inefficiency in reading, spelling or doing maths, generating a deficit with an identifiable effect on the child’s life.

The present cognitive multi-level approach allows interpreting causal relationships at different levels of processing which may be instrumental in understanding previous results as well as making new predictions to be tested in future research. For example, recent research in maths difficulties contrasts a proposal of a deficit in a number module (Landerl et al., 2004; Butterworth, 2005; Wilson and Dehaene, 2007) with the alternative hypothesis of a close link between dyscalculia and a deficiency in visuo-spatial short term memory (Szucs et al., 2013). We propose here that both factors may actually be important, although acting at different levels of processing, the former in terms of numerical “competence”, the second in terms of “processing” (or “performance”) factors required by a typical calculation task. Furthermore, it is possible to make predictions about the possible combined role of competence, performance and acquisition including the presence of partial co-morbidities, i.e., deficits across learning disorders which may be specific of only some sub-components (and indeed may not reach the conventional standards for diagnostic purposes).

The limits of the present study should also be clearly spelled out. We examined the performance of an unselected group of children attending fifth grade on standardized tests of reading, spelling and maths. As we used a relatively large number of tests it was difficult to examine a very large sample; future research should consider the importance to confirm the present findings on a larger sample and also to extend them to other grades so as to support the generality of the conclusions.

As it is well known, performances on reading, spelling and maths tasks are described by continuous distributions and so-called “pathological” performances merely indicate performances lying at the very low ends of such continuous distributions (Protopapas and Parrila, 2018). These considerations suggest the importance of studying an unselected group of children; in particular, one may expect that associations and partial dissociations among key behaviors can be demonstrated both in the “normal” as well as in the “extreme” ranges of performance. Note that this approach is somewhat atypical. For example, models of reading, such as the DRC (Coltheart et al., 2001) or the CDP + (Perry et al., 2007) have been especially developed to account for selective deficits in reading in both acquired and developmental disorders (and similar considerations apply to models of spelling and maths). Still we consider this a good starting point and in future research we will test the multi-level model on children with established mixed deficits in reading, spelling and maths.

## Data Availability Statement

The raw data supporting the conclusions of this article will be made available by the authors, without undue reservation.

## Ethics Statement

The studies involving human participants were reviewed and approved by Comitato Etico – Fondazione Santa Lucia, Rome, Italy. Written informed consent to participate in this study was provided by the participants’ legal guardian/next of kin.

## Author Contributions

PZ, MD, CM, and DS contributed conception and design of the study. MD organized the database and managed software and hardware. CM performed the statistical analysis. MD and CM designed the methodology. PZ wrote the first draft of the manuscript. All authors wrote sections of the manuscript, contributed to manuscript revision, read and approved the submitted version.

## Conflict of Interest

The authors declare that the research was conducted in the absence of any commercial or financial relationships that could be construed as a potential conflict of interest.

## Footnotes

1 https://doi.org/10.1371/journal.pone.0231937.s002
