# Supplementary material for: Testing the Specificity of Predictors of Reading, Spelling and Maths: A New Model of the Association Among Learning Skills Based on Competence, Performance and Acquisition
Source: Front Hum Neurosci. 2020 Dec 7;14:573998. doi: 10.3389/fnhum.2020.573998 (PMC7750359; doi:10.3389/fnhum.2020.573998)
Supplement: Supplementary file 1 [file Table_1.PDF]

Supplementary materials

Table S1. Descriptive statistics for all variables.

Test	Unit of measure	N	Mean	SD	Coefficient of variation	Min	Max	Maximum possible score (closed scales only)	Reliability values
MT reading test (reading time)	time per word (s)	129	0.50	0.11	0.21	0.35	0.95		.85[a]
RAN colours	time per item (s)	129	0.78	0.16	0.21	0.46	1.30		.84[§]
RAN digits	time per item (s)	129	0.50	0.12	0.24	0.32	1.00		.90[§]
Orthographic decoding: Visual-auditory Pseudo-word Matching	number of errors	129	5.1	5.4	1.05	0.0	35.0	90	.60[§]
"Nonna Concetta" Spelling-to-dictation	number of errors	129	2.8	2.2	0.81	0.0	12.0	n.a.	.75[b]
Single Pseudo-word Repetition	number of correct items	129	16.0	4.4	0.27	4.0	25.0	30	.61°
Single Pseudo-word Phonemic Segmentation	number of correct segmentations	129	187.9	23.0	0.12	116.0	223.0	239	.82°
Orthographic Decision	number of errors	129	17.8	7.1	0.40	3.0	33.0	80	.71°
Repetition of Pseudo-word Series	number of correct items	129	18.7	5.2	0.28	4.0	28.0	30	-
Written Arithmetic Calculations	time per item (s)	129	41.8	12.9	0.31	17.5	90.9		.78[c]
Mental and Written Arithmetic Calculations	number of errors	129	2.7	1.8	0.67	0.0	8.0	14	.50[c]
Number Order test	number of errors	129	1.6	1.3	0.82	0.0	7.0	10	.78[d]
Arithmetic Facts test	number of correct items	129	13.2	3.0	0.23	1.0	16.0	16	.71[e]
Computation Strategies test	number of correct items	129	12.6	3.3	0.26	4.0	16.0	16	.78[f]
Symbol Search subtest	number of correct items	129	27.4	4.1	0.15	16.0	39.0	45	.70[g]
Raven's Coloured Progressive Matrices	number of correct items	129	32.1	2.7	0.08	24.0	36.0	36	.80[h]
Forward Span of Numbers	span length (number of digits)	129	5.0	0.9	0.17	4.0	7.0	9	.84[i]
Backward Span of Numbers	span length (number of digits)	129	3.7	0.9	0.26	2.0	7.0	8	-
Verbal Phonemic Fluency test	number of correct items	129	30.6	8.8	0.29	10.0	55.0	n.a.	.83[i]

Legend

Experimental tests: [§] Split-half reliability; ° odd-even reliability. **Standard tests**: n.a. Not applicable; [a] Cornoldi and Colpo, 1998; [b] Marinelli et al., 2016; [c] Cornoldi et al., 2002; [d] the value refers to a factor of "numeric knowledge" which includes this subtest (Cornoldi et al., 2002); [e] Biancardi and Nicoletti, 2004; [f] the value refers to a factor of "numeric facts" which includes this subtest (Cornoldi and Cazzola, 2003); [g] Wechsler, 1986; [h] approximate mean value (Belacchi, C., Scalisi, T. G., Cannoni, E., & Cornoldi, C., 2018; Manuale CPM-Coloured Progressive Matrices. Standardizzazione Italiana, Quarta edizione. Firenze: Giunti Psychometrics); [i] Bisiacchi et al., 2005.

Supplementary materials

Table S2. Intercorrelations table

Pearson correlation matrix	MT reading test (reading time)	MT reading test (accuracy)	RAN colours	RAN digits	Orthographic decoding: Visual-visual Pseudo-word Matching	Orthographic decoding: Visual-auditory Pseudo-word Matching	.Orthographic decoding: Auditory-auditory Pseudo-word Matching	"Nonna Concetta" Spelling-to-dictation	Single Pseudo-word Repetition	Single Pseudo-word Phonemic Segmentation	Orthographic Decision	Repetition of Pseudo-word Series	Written Arithmetic Calculations	.Mental and Written Arithmetic Calculations	Number Order test	Arithmetic Facts test	Computation Strategies test	Symbol Search subtest	Raven's Coloured Progressive Matrices	Forward Span of Numbers	Backward Span of Numbers
MT reading test (reading time)	1	.301**	.394**	.406**	.255**	.443**	.252**	.299**	-.283**	-.336**	.542**	-.429**	.534**	.346**	.244**	-.541**	-.477**	-.291**	-.232**	-.254**	-.249**
MT reading test (accuracy)	.301**	1	.080	.015	.303**	.175*	.082	.287**	-.036	-.263**	.285**	-.301**	.126	.226**	.135	-.185*	-.223*	-.294**	-.235**	-.139	-.262**
RAN colours	.394**	.080	1	.709**	.083	.264**	.080	.063	-.247**	-.100	.118	-.268**	.327**	.175*	.223*	-.423**	-.175*	-.300**	-.244**	-.159	-.118
RAN digits	.406**	.015	.709**	1	.031	.225*	.015	-.037	-.277**	-.042	.012	-.134	.391**	.039	.030	-.470**	-.131	-.312**	-.006	-.220*	-.085
Orthographic decoding: Visual-visual Pseudo-word Matching	.255**	.303**	.083	.031	1	.320**	.373**	.182*	-.043	-.245**	.348**	-.079	.171	.186*	.125	-.289**	-.157	-.147	-.264**	-.075	-.237**
Orthographic decoding: Visual-auditory Pseudo-word Matching	.443**	.175*	.264**	.225*	.320**	1	.641**	.081	-.526**	-.476**	.347**	-.438**	.285**	.278**	.161	-.463**	-.175*	-.241**	-.286**	-.170	-.257**
Orthographic decoding: Auditory-auditory Pseudo-word Matching	.252**	.082	.080	.015	.373**	.641**	1	.072	-.429**	-.361**	.273**	-.287**	.241**	.323**	.157	-.282**	-.140	-.141	-.241**	-.220*	-.135
"Nonna Concetta" Spelling-to-dictation	.299**	.287**	.063	-.037	.182*	.081	.072	1	.031	-.115	.451**	-.347**	.222*	.340**	.367**	-.318**	-.282**	.010	-.237**	-.061	-.130
Single Pseudo-word Repetition	-.283**	-.036	-.247**	-.277**	-.043	-.526**	-.429**	.031	1	.608**	-.230**	.479**	-.206*	-.127	-.198*	.397**	.080	.199*	.286**	.206*	.118
Single Pseudo-word Phonemic Segmentation	-.336**	-.263**	-.100	-.042	-.245**	-.476**	-.361**	-.115	.608**	1	-.296**	.592**	-.106	-.247**	-.245**	.336**	.124	.093	.375**	.206*	.288**
Orthographic Decision	.542**	.285**	.118	.012	.348**	.347**	.273**	.451**	-.230**	-.296**	1	-.413**	.408**	.417**	.495**	-.518**	-.428**	-.195*	-.352**	-.140	-.293**
Repetition of Pseudo-word Series	-.429**	-.301**	-.268**	-.134	-.079	-.438**	-.287**	-.347**	.479**	.592**	-.413**	1	-.185*	-.323**	-.388**	.380**	.271**	.255**	.422**	.249**	.370**
Written Arithmetic Calculations	.534**	.126	.327**	.391**	.171	.285**	.241**	.222*	-.206*	-.106	.408**	-.185*	1	.389**	.281**	-.570**	-.418**	-.298**	-.037	-.197*	-.186*
Mental and Written Arithmetic Calculations	.346**	.226**	.175*	.039	.186*	.278**	.323**	.340**	-.127	-.247**	.417**	-.323**	.389**	1	.372**	-.391**	-.427**	-.154	-.336**	-.147	-.162
Number Order test	.244**	.135	.223*	.030	.125	.161	.157	.367**	-.198*	-.245**	.495**	-.388**	.281**	.372**	1	-.361**	-.388**	-.115	-.448**	.014	-.111
Arithmetic Facts test	-.541**	-.185*	-.423**	-.470**	-.289**	-.463**	-.282**	-.318**	.397**	.336**	-.518**	.380**	-.570**	-.391**	-.361**	1	.352**	.270**	.196*	.177*	.224*
Computation Strategies test	-.477**	-.223*	-.175*	-.131	-.157	-.175*	-.140	-.282**	.080	.124	-.428**	.271**	-.418**	-.427**	-.388**	.352**	1	.357**	.351**	.108	.196*
Symbol Search subtest	-.291**	-.294**	-.300**	-.312**	-.147	-.241**	-.141	.010	.199*	.093	-.195*	.255**	-.298**	-.154	-.115	.270**	.357**	1	.143	.115	.321**
Raven's Coloured Progressive Matrices	-.232**	-.235**	-.244**	-.006	-.264**	-.286**	-.241**	-.237**	.286**	.375**	-.352**	.422**	-.037	-.336**	-.448**	.196*	.351**	.143	1	.087	.275**
Forward Span of Numbers	-.254**	-.139	-.159	-.220*	-.075	-.170	-.220*	-.061	.206*	.206*	-.140	.249**	-.197*	-.147	.014	.177*	.108	.115	.087	1	.231**
Backward Span of Numbers	-.249**	-.262**	-.118	-.085	-.237**	-.257**	-.135	-.130	.118	.288**	-.293**	.370**	-.186*	-.162	-.111	.224*	.196*	.321**	.275**	.231**	1

** p 0.01 (two-tailed); * p 0.05 (two-tailed).

Supplementary materials

Table S3. Supplementary materials.

The table is modelled on Table 1 in the main text except that the effect of the predictors in the "General cognitive factors" model was partialled out over the dependent measures of reading, spelling and calculation. This was carried out by initially calculating multiple regression analyses (with the enter method): in each regression model, the dependent variable was the same as in the previous communality analyses and the predictors of the General cognitive factors model (Raven, Symbol Search, Backward Span, and Verbal Phonemic Fluency) were used as independent variables. Then, the standardized residuals obtained were submitted to communality analyses in order to explore the effect of the predictors in the various models (over both target and non-target behaviour), net of those in the general cognitive factors model.

The table presents the percentage of total variance explained by the reading, spelling, and maths models, once the effect of general cognitive predictors was partialled out. Each specific set of predictors is used to predict the target behaviour as well as all the other non-target behaviours. The variances explained by the "specific" predictions (i.e., reading predicted by predictors in the reading fluency model with general cognitive predictors partialled out, etc.) are marked in bold.

	**Original models**	Reading fluency model	Spelling accuracy model	Calculation (fluency and accuracy) model
**Dependent measures**	Reading fluency	**38.9**	25.1	28.2
	Spelling accuracy	18.0	**26.6**	17.2
	Calculation speed	25.2	14.6	**33.3**
	Calculation accuracy	12.9	13.2	**21.7**
