# Supplementary material for: Testing the Specificity of Predictors of Reading, Spelling and Maths: A New Model of the Association Among Learning Skills Based on Competence, Performance and Acquisition
Source: Front Hum Neurosci. 2020 Dec 7;14:573998. doi: 10.3389/fnhum.2020.573998 (PMC7750359; doi:10.3389/fnhum.2020.573998)
Supplement: Supplementary file 2 [file Image_1.PDF]

Supplementary Figures S1-S4

Vertical bars report the 95% confidence limits of the coefficients of communality presented in Table 3. These estimates were obtained as accelerated bootstrap confidence intervals produced over 1,000 iterations (Nimon and Oswald, 2013). In each figure, the behaviour considered is indicated in the top (Reading, Spelling, Calculation), and the top graph refers to data for the model based on specific predictors, i.e. Reading predicted by Reading predictors, Spelling predicted by Spelling predictors, and Calculation predicted by Calculation predictors (Zoccolotti et al., 2020). The middle and the bottom graphs refer to data obtained testing the cross-over predictors efficacy. For example: Figure S1 middle graph reports coefficients of communality for Reading predicted by predictors in the Spelling model, and Figure S1 bottom graph reports coefficients of communality for Reading predicted by predictors in the Calculation model.

READING

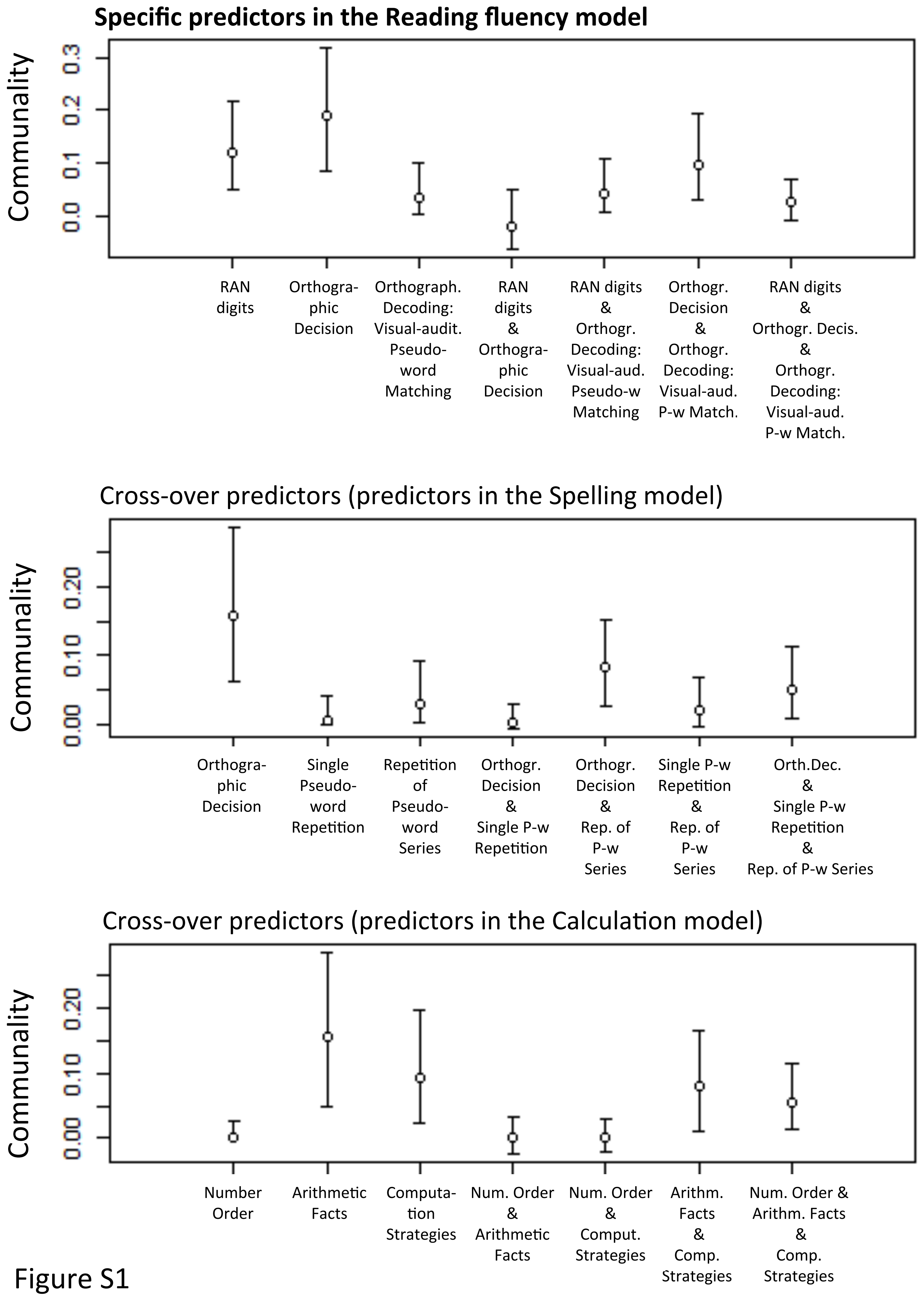

Figure S1

SPELLING

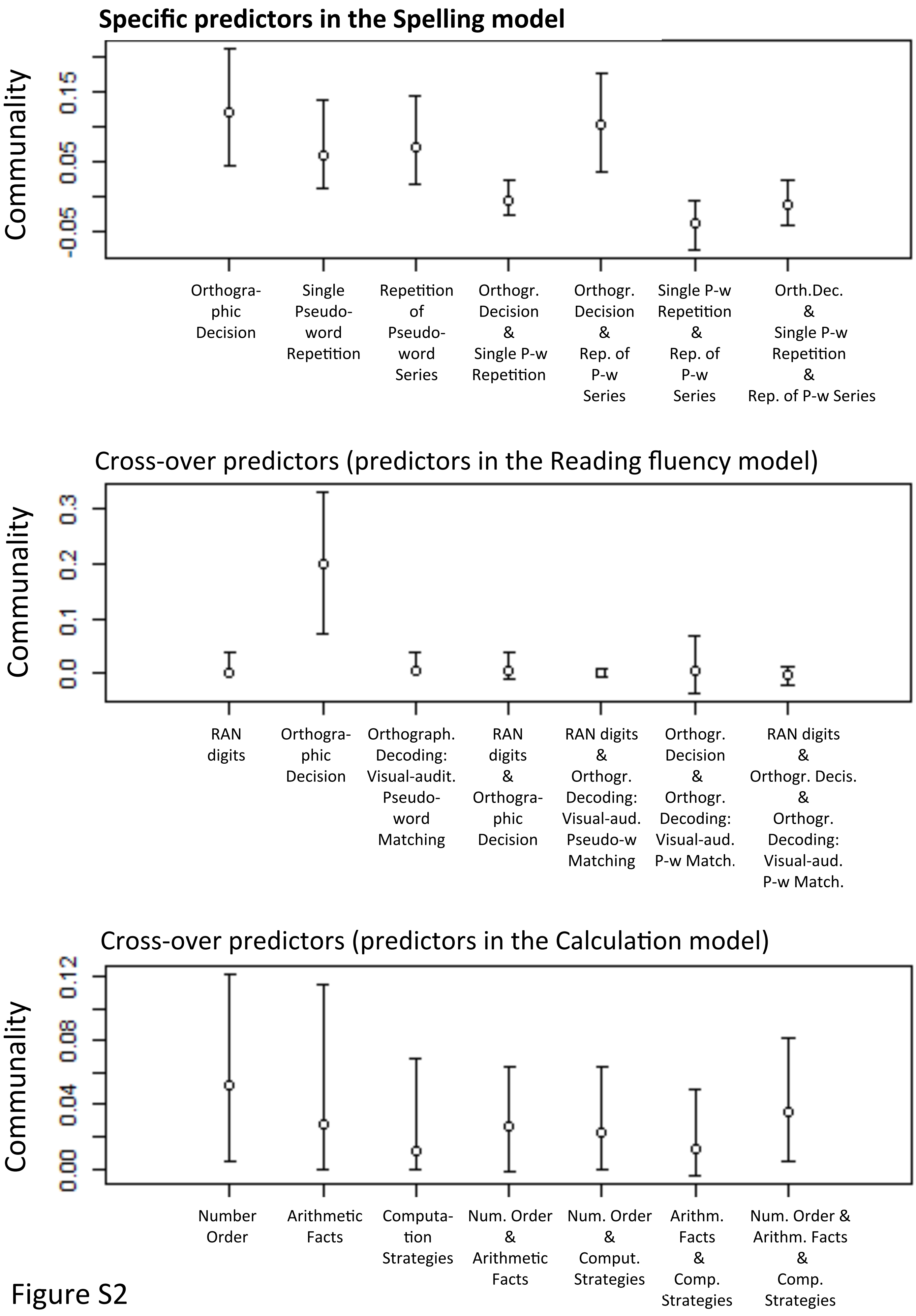

Figure S2

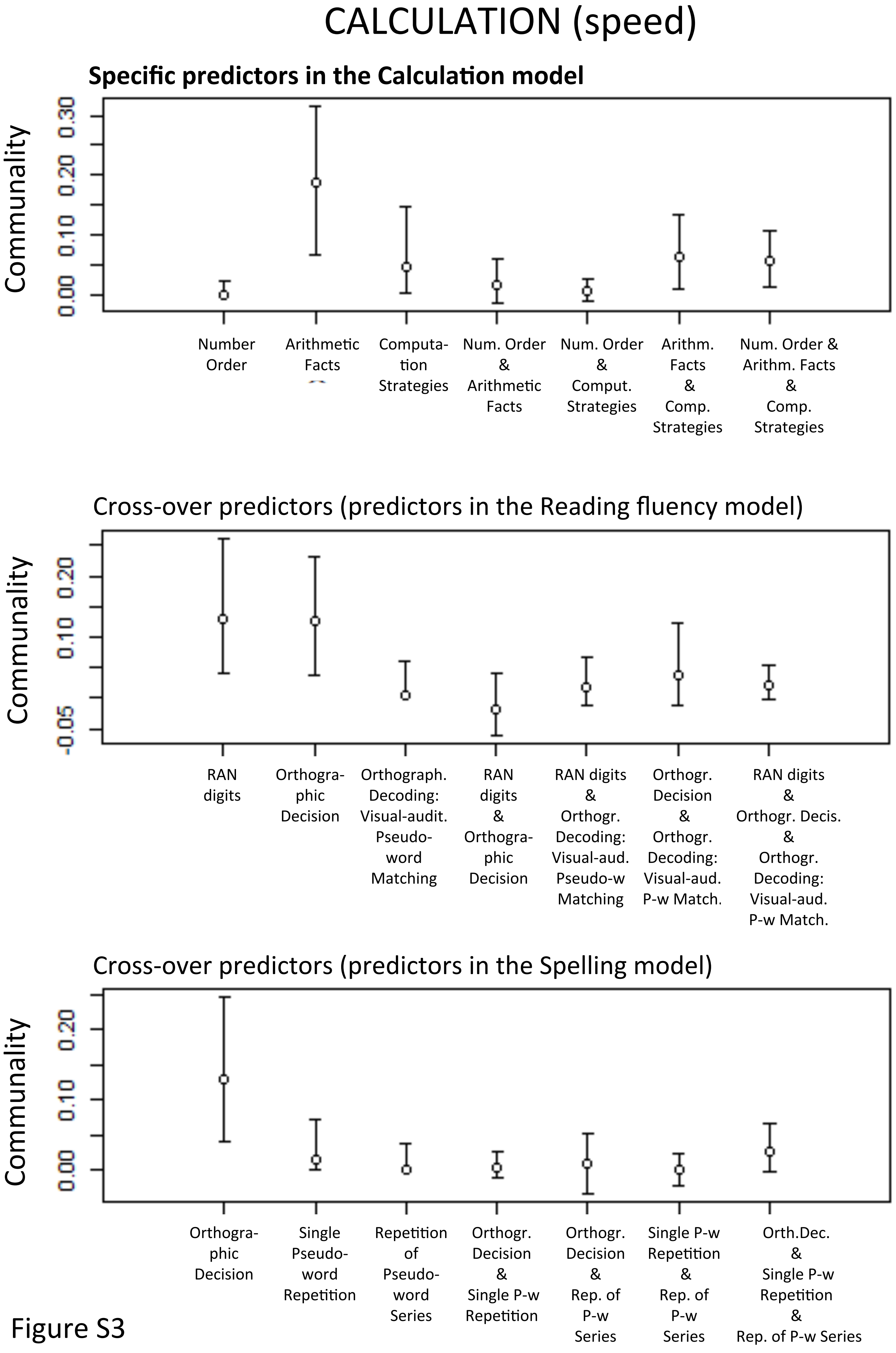
CALCULATION (speed)
Specific predictors in the Calculation model
Communality
0.00
0.10
0.20
0.30
Number Order
Arithmetic Facts
Computa-tion Strategies
Num. Order & Arithmetic Facts
Num. Order & Comput. Strategies
Arithm. Facts & Comp. Strategies
Num. Order & Arithm. Facts & Comp. Strategies
Cross-over predictors (predictors in the Reading fluency model)
Communality
-0.05
0.10
0.20
RAN digits
Orthogra-phic Decision
Orthograph. Decoding: Visual-audit. Pseudo-word Matching
RAN digits & Orthogra-phic Decision
RAN digits & Orthogr. Decoding: Visual-aud. Pseudo-w Matching
Orthogr. Decision & Orthogr. Decoding: Visual-aud. P-w Match.
RAN digits & Orthogr. Decis. & Orthogr. Decoding: Visual-aud. P-w Match.
Cross-over predictors (predictors in the Spelling model)
Communality
0.00
0.10
0.20
Orthogra-phic Decision
Single Pseudo-word Repetition
Repetition of Pseudo-word Series
Orthogr. Decision & Single P-w Repetition
Orthogr. Decision & Rep. of P-w Series
Single P-w Repetition & Rep. of P-w Series
Orth.Dec. & Single P-w Repetition & Rep. of P-w Series

Figure S3

CALCULATION (accuracy)

Specific predictors in the Calculation model

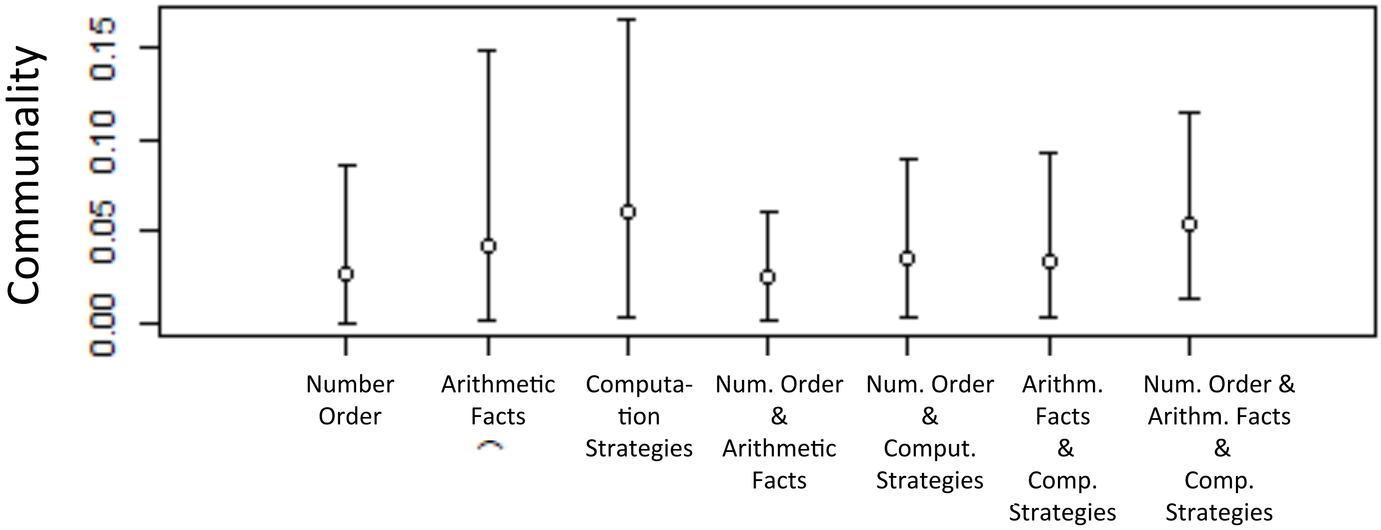

Cross-over predictors (predictors in the Reading fluency model)

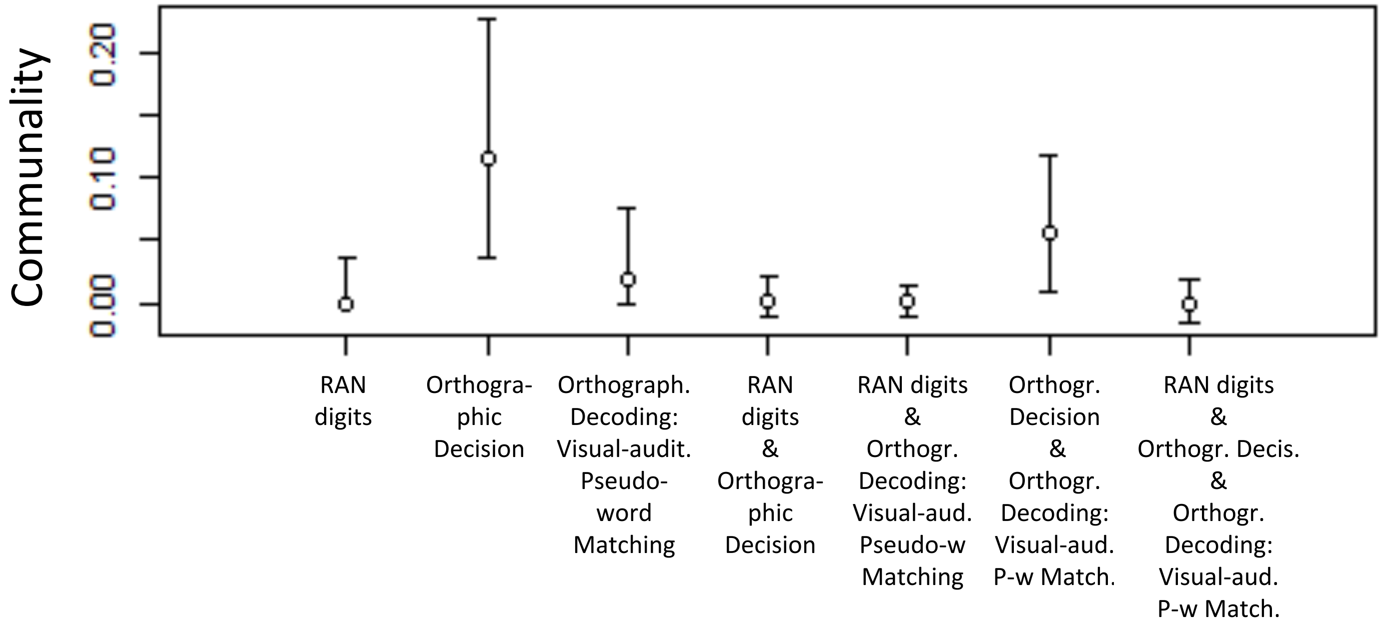

Cross-over predictors (predictors in the Spelling model)

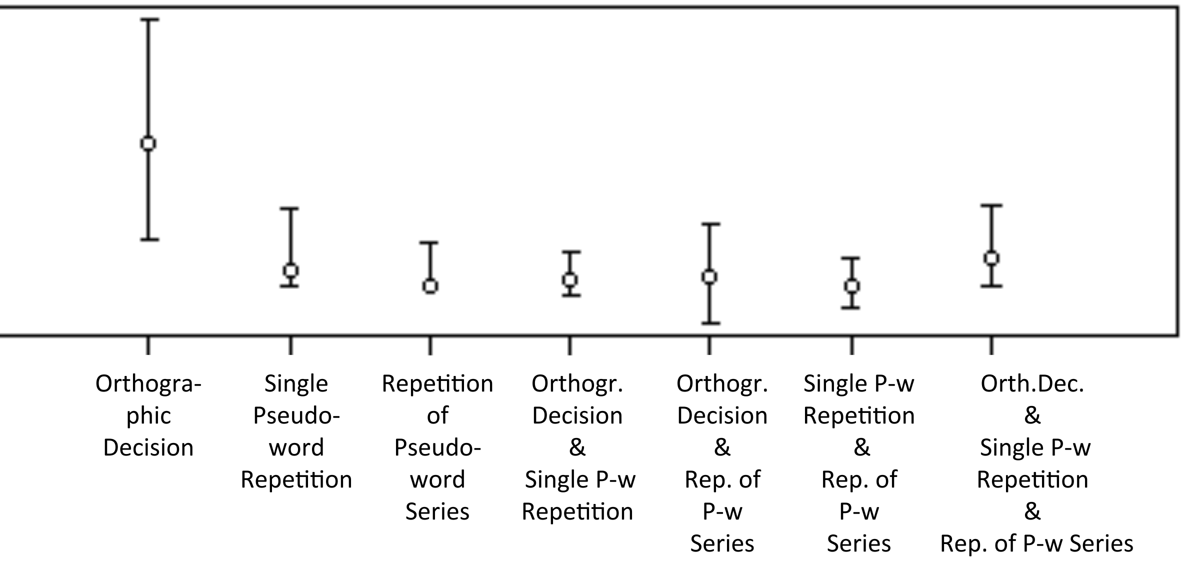

Figure S4
